# Ceramides as Biomarkers of Risk Prediction and Prognosis in Cardiovascular and Other Diseases: Are We There Yet?

**DOI:** 10.3390/ijms27219605

**Published:** 2026-10-27

**Authors:** Thansi Garikipati, Priya Dubey, Dylan Work, Sanidhya Oli, Dhruv Akhilesh Sharma, Subroto Chatterjee

**Affiliations:** Cardiovascular Innovation Laboratory, The Helen B. Taussig Center, Division of Cardiology, Department of Pediatrics, School of Medicine, Johns Hopkins University, Baltimore, MD 21287, USA; tgariki1@jh.edu (T.G.); pdubey5@jh.edu (P.D.); dwork1@jh.edu (D.W.); soli1@jh.edu (S.O.); dsharm37@jh.edu (D.A.S.)

**Keywords:** ceramides, sphingolipids, biomarkers, cardiovascular disease, CERT2, lipidomics, risk prediction, prognosis

## Abstract

Cardiovascular disease (CVD) is the leading cause of death worldwide, yet roughly 70% of major adverse cardiovascular events occur in statin-treated patients with optimized Low-density lipoprotein cholesterol (LDL-C), a gap that cholesterol-based risk panels do not close. Ceramides are bioactive sphingolipids whose plasma species and ratios carry risk information absent from standard lipid panels, and ceramide-based scores (CERT and CERT2) are already in clinical use. Whether this value extends beyond CVD is unclear. We reviewed ceramide species and ratios as biomarkers for disease prediction, prognosis, and disease activity across cardiovascular, metabolic, autoimmune rheumatic, inflammatory bowel, pulmonary, kidney, and oncologic contexts, searching PubMed and Scopus from January 1990 to June 2026 and extending the search for the historical literature. In CVD, CERT and CERT2 predicted cardiovascular events and mortality independently of LDL cholesterol, including in statin-treated and kidney disease populations. In metabolic and autoimmune rheumatic disease, ceramide profiles tracked cardiovascular risk and disease activity. In inflammatory bowel disease, chronic obstructive pulmonary disease, and most cancers, the evidence is mechanistically strong but limited to single-cohort or observational studies. Ceramides are emerging biomarkers that complement, not replace, established risk tools. Broader clinical use requires prospective validation, diverse cohorts, standardized assays, and demonstrated cost-effectiveness.

## 1. Introduction

Ceramides are an incredibly varied and diverse group of sphingolipids composed of a sphingoid base linked to a fatty acyl chain of varying length and saturation [1]. Once considered basic structural components of cellular membranes, they are now recognized as active lipids that regulate a wide range of cellular processes, including apoptosis, inflammation, insulin signaling, mitochondrial function, and stress responses [1,2]. These functions position ceramides at the intersection of metabolic regulation and disease pathogenesis, particularly in conditions characterized by chronic inflammation and lipotoxic stress [3].

As mass-spectrometry lipidomics have advanced, simply totaling ceramides has given way to more specific, chain-length-oriented detection of ceramides [4,5]. In fact, it is ceramide molecular species and the ratios between long-chain and very-long-chain ceramides that encode biologically meaningful information usually invisible to traditional lipid measurements like low- density lipoprotein cholesterol (LDL-C) or triglycerides panels [6,7]. These discoveries have paralleled the growing evidence that ceramides are actively involved in lipid metabolism [3].

A multitude of large cohort studies have demonstrated that a distinct set of ceramide species, Cer(16:0), Cer(18:0), and Cer(24:1), are strongly predictive of major adverse cardiovascular events (MACE), cardiovascular mortality, and all-cause mortality, often outperforming traditional risk factors like cholesterol [6,8,9]. This predictive strength becomes strongest when these ceramides are normalized to protective very-long-chain species like Cer(24:0), highlighting the pathological nature of certain ceramides instead of focusing on lipid excess [6,8,9].

Yet, ceramide dysregulation spills far beyond cardiovascular disease. Altered ceramide profiles have been implicated in metabolic diseases that strongly amplify Cardiovascular Disease (CVD) risk, including type 2 diabetes, obesity, and metabolic syndrome [6,10], as well as in inflammatory and immune-mediated disorders such as inflammatory bowel disease (IBD), rheumatoid arthritis, systemic lupus erythematosus, chronic obstructive pulmonary disease, and oncological diseases [11,12,13,14,15]. One factor stands out: the ratio of long-chain, pro-inflammatory ceramides like Cer(16:0) and Cer(18:0) to very-long-chain species like Cer(24:0), which points to a marker of cellular stress and disarray [1].

In this review, we aim to distinguish between the roles a circulating biomarker can have, since the evidence for ceramides differs significantly between them. In this text, a diagnostic biomarker identifies disease/disease subtype at the time of testing, whereas risk prediction/susceptibility biomarkers aim to estimate the probability that an unaffected individual will develop a disease or adverse event. A prognostic biomarker estimates likelihoods of future clinical and health outcomes in diseased individuals, compared with predictive biomarkers which identify individuals more likely to benefit from certain therapies [16]. Most clinical evidence for ceramide-based scores, including CERT1 and CERT2, concerns risk prediction and prognosis rather than diagnosis, and we use the term “diagnostic” only where a study evaluated ceramides for identifying a present condition.

While the evidence continues to grow regarding ceramides and their role in disease, many important questions remain unresolved. This review sets out to synthesize and analyze the evidence on ceramides for a comprehensive set of cardiovascular and related non-cardiovascular diseases, and to ask three questions of it. Are ceramides truly independent markers of prognosis and disease progression, or do they simply reflect generalized dyslipidemia and lipid excess? Can ceramide ratios offer incremental value beyond established clinical risk scores? Finally, are we close enough to clinical implementation to justify ceramides’ routine use across diseases, or is their current utility confined to cardiovascular risk prediction?

## 2. Methods

### 2.1. Search Strategy

The literature search was conducted through targeted database searches of PubMed and Scopus between 1 January 1990 and 30 June 2026, combining free-text terms for ceramides and sphingolipids with terms for each of 14 disease categories (cardiovascular disease, cardiovascular risk prediction and CERT scores, heart failure and cardiomyopathy, autoimmune rheumatic diseases, rheumatoid arthritis, systemic lupus erythematosus, metabolic disease, type 2 diabetes and insulin resistance, non-alcoholic fatty liver disease, cancer, therapeutic resistance and ceramide metabolism, inflammatory bowel disease, COPD and pulmonary disease, and chronic kidney disease). The complete search strings and the number of records retrieved for each category are provided in Table S1. Reference lists of included articles and recent reviews were also hand-searched. Because Section 3.2 traces the historical development of the field, we additionally performed a separate search without date limits for foundational studies on sphingolipid structure and ceramide signaling; records from this search are reported separately in Figure S1.

### 2.2. Structure and Criteria for Eligibility

We included peer-reviewed studies focused on ceramides in humans or mechanistic models that reported species-level ceramide or ceramide-score data with adequate methodological detail. Conference abstracts, non-peer-reviewed reports, duplicate datasets and studies without species-level ceramide data were excluded. For the clinical sections, priority was given to cohort and lipidomic studies with validated quantification, clearly defined methods and adequate sample sizes.

A PRISMA workflow was not utilized because of the review’s narrative design; however, the papers selected were primarily cohort studies and lipidomic analyses, with emphasis on validated lipidomic quantifications, clearly defined methodology and processes, and sufficient sample sizes. The limitations of the lack of a PRISMA workflow in this review are acknowledged.

### 2.3. Study Selection

The 14 disease-category searches retrieved 25,395 records, and the background and historical search retrieved 131 records, a total of 25,526 records (Table S1). Records were screened by title and abstract against the criteria in Section 2.2, with the full text reviewed where eligibility was unclear. Of the 25,526 records, 25,345 were excluded, including records retrieved by more than one category search, and 181 articles were included in the review: 147 from the disease-category searches and 34 from the background and historical search. Each included article is counted once, in the category in which it is cited most often (Table S1). The selection process is summarized in Figure S1.

### 2.4. Classification of Evidence Strength

For each disease, the clinical evidence for every ceramide species, ratio, or score was categorized into one of six levels of translational readiness (assigned by T.G. and reviewed by S.C.). These tiers reflect, informally, how widely a marker has been replicated and applied in clinical settings. They do not measure study quality, risk of bias, or formal certainty (such as GRADE), and a higher tier does not necessarily mean testing improves patient outcomes.

- Dark Green (Clinically Deployed): Available as an active clinical lab test for the specific indication and validated across multiple independent cohorts (e.g., CERT1 and CERT2 for cardiovascular risk).
- Light Green (Replicated Human Evidence): Associated with the clinical endpoint in at least two independent patient/population cohorts, or in one cohort plus a post hoc trial analysis, without active clinical deployment.
- Yellow (Single-Cohort Human Evidence): Tied to the clinical endpoint (adjusted for standard clinical factors where noted) in one adequately sized cohort or pooled report, lacking independent replication.
- Orange (Preliminary Human Evidence): Based on small cross-sectional or case–control studies, tissue-only measurements, associations that lost statistical significance after adjustment, or unvalidated multi-marker panels.
- Red (Mechanistic or Preclinical Only): Derived strictly from cell or animal models, with no patient data relevant to the disease.
- Gray (No Evidence): No relevant studies found in the literature search.

## 3. Background

### 3.1. Biochemistry

**Figure 1:**
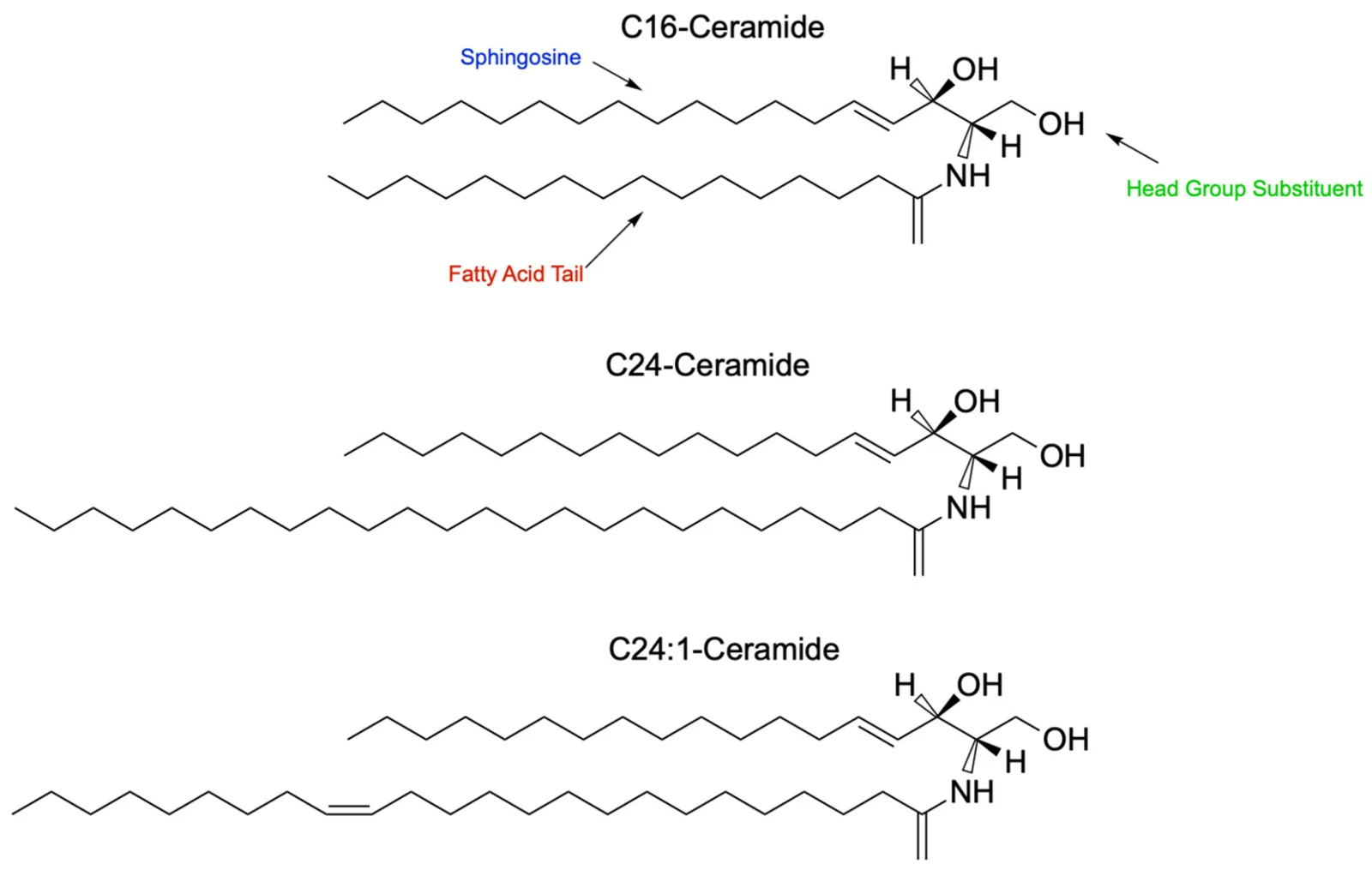
The makeup of ceramide. Ceramides consist of a sphingosine attached to a fatty acid tail that is variable in length, with the number of carbons in the tail denoted. Here, 3 ceramides of varying tail length and double-bond counts are depicted.

Ceramides are sphingolipids composed of a long-chain sphingoid base, usually sphingosine, an 18-carbon amino alcohol, linked via an amide bond to a fatty acid chain that typically ranges from 14 to 36 carbons (Figure 1). This linkage happens via an N-acylation reaction, catalyzed by ceramide synthase (CerS) enzymes and reversed by ceramidases, putting ceramide as an important intermediate within the sphingolipid networks and metabolic crossroads. Ceramides are a precursor to sphingomyelins, glucosylceramides, and sphingosine-1-phosphate (S1P) [1,17,18,19,20].

Structurally, ceramides comprise a small polar head group and two hydrophobic chains, giving the sphingolipid an amphipathic, conical shape; this geometry promotes membrane curvature, stabilizes lipid raft microdomains, and enables ceramide to function as a second messenger in apoptosis, differentiation, and inflammatory signaling; these utilities and signaling roles were recognized following key work by Hannun, Obeid, and Kolesnick in the late 1980s and 1990s [1,17,19,20].

**Figure 2:**
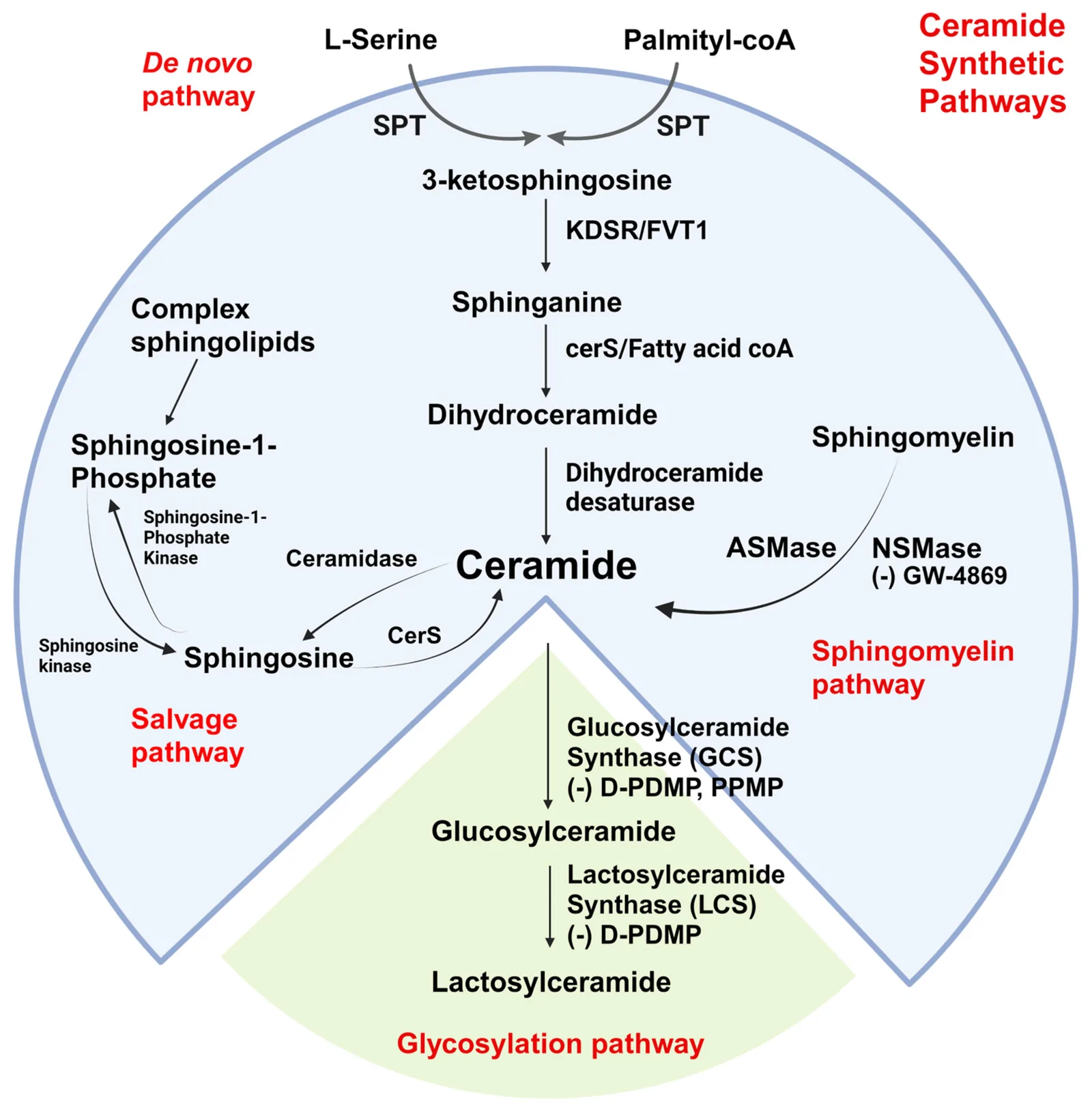
The three predominant ceramide metabolic pathways (de novo, sphingomyelin, and salvage pathway) as mentioned in Section 3 are shown here. The downstream modification of ceramide to glucosylceramide and lactosylceramide is also shown. Created in https://BioRender.com.

**Figure 3:**
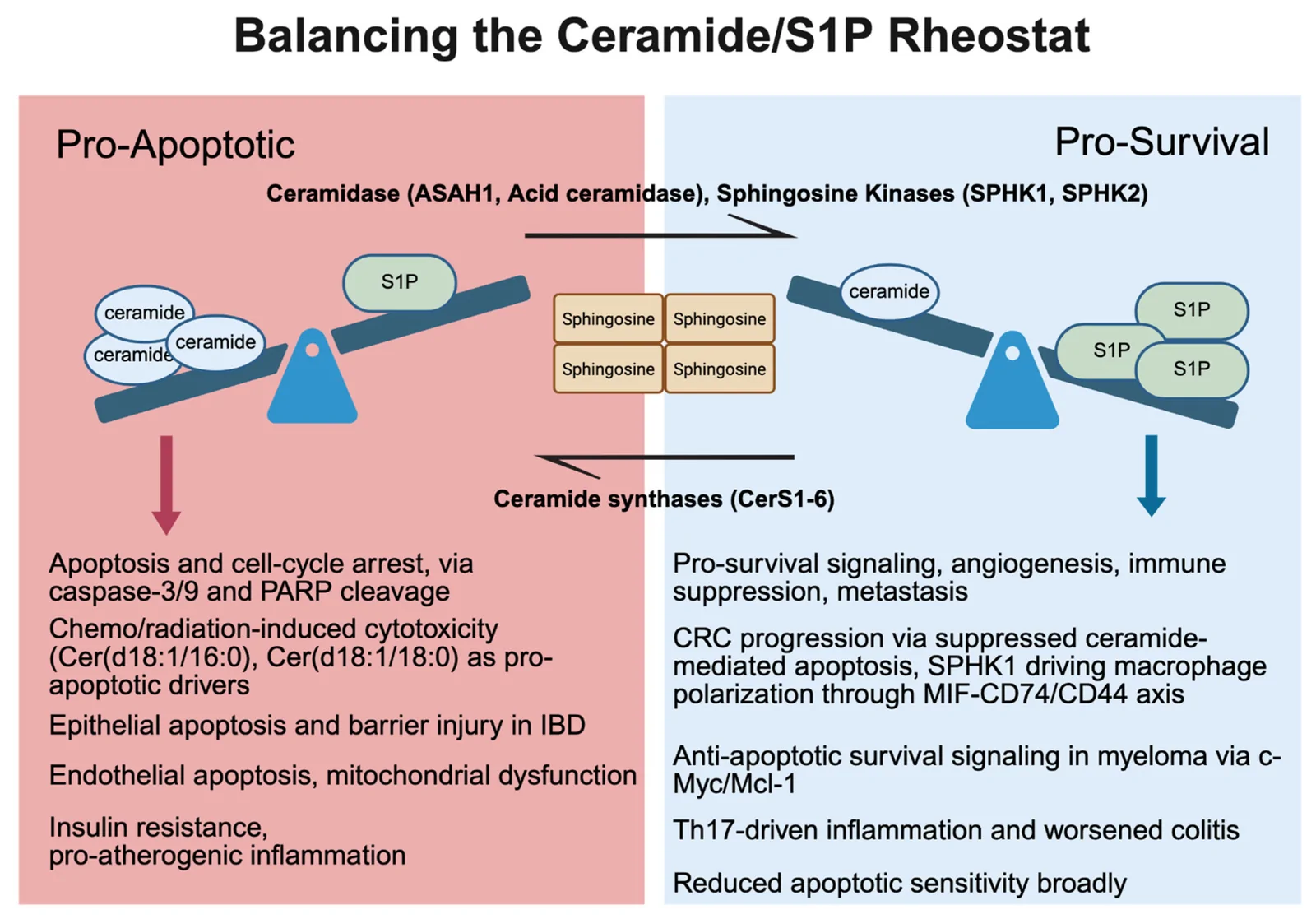
The ceramide/sphingosine-1-phosphate (S1P) rheostat. Ceramidases (ASAH1) and sphingosine kinases (SPHK1/2) convert ceramide to sphingosine-1-phosphate (S1P), shifting cells toward survival, while ceramide synthases (CerS1–6) convert sphingosine back to ceramide, shifting cells toward apoptosis. Ceramide accumulation promotes apoptosis, therapy-induced cytotoxicity, and inflammatory and metabolic injury, whereas S1P accumulation promotes survival signaling, angiogenesis, immune suppression, and metastasis. Created in https://BioRender.com.

Ceramide production and availability are regulated through three synthetic pathways (Figure 2). First is the de novo pathway, the dominant route of ceramide production, initiated in the endoplasmic reticulum with the condensation of L-serine and palmitoyl-CoA, catalyzed by serine palmitoyltransferase (SPT), to yield 3-ketosphingosine; subsequent reduction and acylation by CerS generate dihydroceramide, which is then desaturated to ceramide by dihydroceramide desaturase [17]. In contrast, the sphingomyelinase pathway is used as a rapid and stress-responsive source of ceramide; neutral sphingomyelinase (NSMase) and acid sphingomyelinase (ASMase) hydrolyze sphingomyelin at the plasma membrane and within lysosomes, respectively, thereby releasing ceramide in response to inflammatory cytokines, oxidative stress, and receptor-mediated signaling events [17]. Finally, the salvage pathway serves as a recycling process to form ceramides by converting long-chain sphingoid bases (from degradation of complex sphingolipids) back into ceramide via CerS-mediated re-acylation; this pathway also produces sphingosine and S1P as intermediate metabolites [1,17,19,20]; the balance between ceramide and S1P is illustrated in Figure 3.

An important and increasingly significant aspect of ceramide biology lies in the functional specificity of the six mammalian CerS isoforms (CerS1–6); each isoform exhibits distinct fatty acid chain-length preferences, and this specificity directly shapes the biological and prognostic properties of the ceramide species produced (Table 1) [18,21]. CerS5 and CerS6 preferentially incorporate C16:0 fatty acids to generate Cer(16:0), whereas CerS1 produces Cer(18:0); these long-chain species are associated with mitochondrial dysfunction, insulin resistance, endothelial apoptosis, and pro-atherogenic inflammation [2,18,22]. By contrast, CerS2 synthesizes very-long-chain species such as Cer(22:0) and Cer(24:0); these species are increasingly recognized as structurally and functionally protective, contributing to membrane integrity and potentially mitigating the lipotoxic effects of shorter-chain ceramides [8,18,23]. This chain-length-dependent biology forms the basis for the biomarker framework seen in CERT and CERT2; beyond total ceramide quantities, the balance between risk-associated long-chain species (Cer(16:0), Cer(18:0)) and very-long-chain species (Cer(22:0), Cer(24:0)) adds clinically meaningful information for understanding and codifying cardiovascular and metabolic risk [20,22,23,24,25,26]. However, these labels are generalizations rather than fixed properties of each species, since the biological effects of ceramides depend on tissue, cell type, subcellular localization, and metabolic context [1]. For example, Cer(24:1), although a very-long-chain species made mainly by CerS2, behaves as a risk-associated species in cardiovascular cohorts [6], while the ultra-long-chain Cer(d18:1/26:0) promotes colorectal cancer growth rather than protecting against it (Section 10.3). Throughout this review, “harmful” and “protective” therefore describe the associations reported in the cited studies rather than universal effects.

Chain-length preferences are summarized from [18,21]. Ratios of risk-associated species to Cer(d18:1/24:0), together with absolute concentrations (CERT1) or ceramide/phosphatidylcholine ratios (CERT2), underlie the CERT scores [9,31].

Finally, ceramide dysregulation should not be seen as just a sole consequence of increased synthesis or impaired catabolism but instead as pathological changes and remodeling within the sphingolipid metabolic crossroads. This can be observed in shifts in CerS isoform activity and sphingomyelinase-driven hydrolysis favoring accumulation of long-chain ceramides and the depletion of very-long-chain species alongside overactivation of SPT-mediated de novo synthesis, as seen with saturated fatty acid excess, obesity, and insulin resistance. These lipid-based changes promote lipotoxic ceramide accumulation in the liver, skeletal muscle, and myocardium [18]. Similarly, increased NSMase and ASMase activity in inflammatory and ischemic states leads to rapid ceramide generation at cellular membranes; this significantly increases oxidative stress, disrupts Akt-mediated insulin signaling, and activates apoptotic pathways [17]. Taken together, these processes demonstrate that specific ceramide species, and their interconnected enzymatic synthetic systems, are highly involved in inflammation, metabolic dysfunction, and cardiovascular disease. This provides priority for understanding and quantifying ceramide species ratios, in addition to absolute concentrations, in risk stratification and clinical applications across disease contexts [3,19,20].

### 3.2. Historical Perspectives

The historical perspective on ceramides and disease has evolved significantly over the past century, from viewing them as inert structural components of cellular membranes to recognizing them as potent, bioactive signaling molecules that regulate inflammation, apoptosis, metabolism, and disease progression. This evolution spans four distinct phases: structural discovery, mechanistic signaling, lipidomic revolution, and clinical translation.

Sphingolipids were first isolated as major components of brain tissue in 1884 by Johann Ludwig Wilhelm Thudichum [32], seen primarily in cell membranes. He named the long-chain amino alcohol backbone of the nitrogen-containing phospholipids as “sphingosine,” in reference to the sphinx-like feature of the unique, new compound. For decades, sphingolipids were believed to function as the primary structural components of cellular membranes. In 1947, sphingosine was formally confirmed as 2S,3R,4E-2-aminooctadec-4-ene-1,3-diol when Herbert Carter and colleagues performed the definitive structural characterization and proposed the formal designation of sphingosine-derived lipids as “sphingolipids” [33]. For the next couple of decades, ceramides were understood primarily as membrane components that mainly contributed to the structural integrity and impermeability of lipid bilayers. Their work established the biochemical framework of sphingolipid research today.

This understanding of ceramides as inert membrane components changed in the 1980s and 1990s, when landmark studies established ceramides as a key intracellular second messenger. Sphingomyelin hydrolysis and subsequent ceramide formation were implicated in programmed cell death through the regulation of tumor necrosis factor-alpha (TNF-α) on cell growth and differentiation [34,35,36,37,38].

This was followed by Kolesnick and Golde in 1994, who found that activation of the sphingomyelin pathway, heightening the ceramide concentration, accelerated DNA fragmentation, thus furthering cell death and apoptotic DNA damage [37,39]. Hannun’s findings in 1996 then emphasized ceramide’s key role in signal transduction and cell regulation of apoptosis, cell cycle arrest, and senescence through involvement in intracellular stress response pathways [36].

Subsequent studies demonstrated that ceramide effects are highly context-dependent and vary with ceramide species, subcellular localization, and metabolic changes [40]. This complexity has interfered with direct interventional efforts to utilize ceramides as anticancer agents [41]. Tumor cells were found to upregulate ceramide-metabolizing enzymes, principally glucosylceramide synthase and acid ceramidase, reducing ceramide-induced apoptotic signals and driving therapeutic resistance [42,43].

The emergence of electrospray ionization tandem mass spectrometry and its application to plasma lipidomics in the 2000s was the technical shift that moved ceramide research from a mechanistic cell-biology discipline into a clinically usable field. For the first time, investigators could quantify discrete ceramide molecular species, Cer(d18:1/16:0), Cer(d18:1/18:0), Cer(d18:1/24:0), and others, at nanomolar concentrations in patient plasma, with enough resolution to detect meaningful differences between species that differ only in acyl chain length, a resolution not accessible to earlier thin-layer chromatography or HPLC-based methods.

Laaksonen and colleagues provided the first large-scale clinical evidence in 2016, showing in a prospective cohort that a panel of plasma ceramide species independently predicted major adverse cardiac events beyond conventional risk factors [6]. Peterson and colleagues then validated these findings across over 5700 participants from the Framingham Heart Study and the Study of Health in Pomerania, demonstrating that Cer(d18:1/24:0)/Cer(d18:1/16:0) ratios were inversely associated with incident coronary heart disease, heart failure, and all-cause mortality [8,44]. Meeusen and colleagues at the Mayo Clinic characterized the clinical correlates of plasma ceramide elevation, showing independent associations with obesity, insulin resistance, and diabetes mellitus, and finding that atherosclerotic plaques are highly enriched in ceramides, with ceramide accumulation potentially accelerating atherogenesis by promoting LDL infiltration into the endothelium and aggregation within the arterial intima [10,45]. This body of work led to the development of the Ceramide Risk Score, formalized as CERT1 and subsequently refined as CERT2 by Hilvo and colleagues, who incorporated ceramide-to-phospholipid ratios for improved predictive performance [9]. CERT2 was commercially deployed through Zora Biosciences and licensed to the Mayo Clinic and Cleveland HeartLab, marking ceramides’ entry into clinical cardiovascular risk stratification. Specific long-chain ceramides, Cer(16:0), Cer(18:0), and Cer(24:1), have been shown to predict major adverse cardiac events with a strength that outperforms LDL cholesterol [2], and the application of ceramide panels is beginning to extend beyond the cardiometabolic range, with tools such as the PCPro screener exploring ceramide-based prognosis prediction in prostate cancer [46].

Overall, the history of ceramide research demonstrates a fundamental shift from structural lipid biology to precision lipidomics. This evolution has matured into an understanding of distinct ceramide molecular species and their ratios, which now function as both pathological mediators and clinically actionable biomarkers across various disease states.

## 4. Ceramides as Predictors and Markers of CVD Risk

### 4.1. Do Ceramide Levels Alone Predict CVD Events?

Current literature points toward the fact that the predictive strength of circulating ceramides is greatest when specific molecular species, and the ratios between them, are considered rather than total ceramide content. The first major study pushing this idea, by Laaksonen and colleagues, showed in patients with stable coronary artery disease and acute coronary syndromes that plasma concentrations of Cer(d18:1/16:0), Cer(d18:1/18:0), and Cer(d18:1/24:1) were associated with cardiovascular death while Cer(d18:1/24:0) was not, and that their ratios to Cer(d18:1/24:0) were also strongly associated with these outcomes [6]. Meeusen and colleagues reported similar associations with major adverse cardiovascular events in patients undergoing coronary angiography [10]. This approach laid the foundation for Zora Biosciences’ development and patent of the Coronary Event Risk Test (CERT1), which combines the three absolute concentrations with their three ratios to Cer(d18:1/24:0) (Table 2) [6,31]. Absolute concentrations therefore do carry prognostic information, and the ratios add to it rather than replace it, plausibly because they partly normalize for overall lipoprotein and sphingolipid burden. The test, alongside its updated version CERT2, was eventually licensed and used in clinical settings like Mayo Clinic and Cleveland HeartLab [6,31,47].

Other papers have identified elevated ceramides as a unified risk factor when researchers began looking at which ceramides were high relative to others. A study examining the Framingham Heart cohort found that individuals with low levels of very-long-chain ceramides like Cer(24:0) and Cer(22:0) compared to Cer(16:0) actually had a higher risk of Coronary Heart Disease (CHD), heart failure, and overall mortality even though these were otherwise healthy, low-risk adults [8].

Newer research points toward the type of ceramide abundance being more predictive, in comparison to just overall ceramide abundance. This paper analyzed a large, healthy, community-based cohort without usual traditional risk factors (non-smokers without major diseases/comorbidities) [50]. The investigators found that the ratios of very-long-chain to long-chain ceramides, specifically Cer(24:0)/Cer(16:0) and Cer(22:0)/Cer(16:0), reveal a strong inverse relationship with mortality and cardiovascular risk, defying what the common literature consensus seemed to believe. The Framingham Heart Study dataset demonstrated that individuals with the lowest ratios of very-long-chain to long-chain ceramides surprisingly had the highest risk for coronary heart disease, heart failure, and general mortality, contradicting earlier beliefs regarding ceramide correlation with cardiovascular risk. In fact, ceramide metabolism is impacted by what is known as pathological remodeling, where protective very-long-chain species (C22:0, C24:0) decrease and pro-atherogenic long-chain species (C16:0) increase, driving up the risk-enhancing lipid ratio. This underscored the need for scientists to look at the predictive value not merely through a ceramide concentration lens, but instead through the ratios between distinct ceramide types and lengths.

As evidence grew that specific ceramide ratios independently predict cardiovascular event risk, Hilvo and colleagues developed CERT2 in the Western Norway Coronary Angiography Cohort (WECAC; *n* = 3789) and validated it in the LIPID trial (*n* = 5991) and the KAROLA cohort (*n* = 1023) [9]. While CERT1 focused on ceramides and ceramide ratios, CERT2 integrates ceramides with certain phosphatidylcholines (PCs): its four lipid components are the ratios Cer(d18:1/24:1)/Cer(d18:1/24:0, Cer(d18:1/16:0)/PC(16:0/22:5), and Cer(d18:1/18:0)/PC(14:0/22:6), and the concentration of PC(16:0/16:0) (Table 2) [9]. Each component is scored by quartile to give a total score of 0–12, which is divided into four risk categories [9,31]. Importantly, age and sex are not part of the lipid score itself; they were added only in the extended prognostic model. In WECAC, CERT2 combined with age and sex reached a C-index of 0.76 for cardiovascular death but 0.65 for composite cardiovascular events (cardiovascular death, MI, and stroke) [9], and CERT2 was more strongly associated with cardiovascular death than CERT1 (hazard ratio per standard deviation 1.50 vs. 1.27 in WECAC) [31]. CERT2 therefore discriminates cardiovascular mortality reasonably well in patients with established coronary disease, while its discrimination for broader composite endpoints is more modest.

Furthermore, the paper highlighted CERT2’s bridging of an important clinical gap: heterogeneous risk, which traditional clinical metrics like HDL/LDL lipid profiles fail to measure. Generally, patient populations at very high risk, like individuals with a 10-year risk of death being +15–20%, often fail proper recognition because of low identification of this risk by traditional testing measures. Yet CERT2 is effective in identifying very-high-risk patient populations through incorporating ceramide and phosphatidylcholine measures alongside traditional risk factors. This demonstrates the need for ceramides to be incorporated alongside other molecules for assessing CVD risk, and to consider other types of markers like phospholipids.

How does ceramide testing hold up against other accepted methods of CVD risk testing like LDL cholesterol, Systemic Coronary Risk Evaluation 2 (SCORE2), and Atherosclerotic Cardiovascular Disease (ASCVD) tests? For predictive accuracy, the CERT2 risk score has a higher univariate c-index when compared to SCORE2 and ASCVD, showing CERT2’s performance by itself is the best out of most traditional CVD tests [48]. It outperformed LDL testing when it came to CV death, all-cause death, and MACE (Major Adverse Cardiovascular Events), and combining CERT2 and LDL testing created a more powerful predictive model, especially when including traditional risk factors like BMI and smoking status [48].

Although CERT2 has demonstrated promising predictive performance for cardiovascular outcomes, its incremental value beyond established risk assessment models requires further evaluation. Studies comparing CERT2 with conventional approaches, including SCORE2, ASCVD risk estimation, and LDL cholesterol measurements, have reported favorable discrimination for certain cardiovascular outcomes [48]. However, superior performance in univariate analyses does not necessarily establish that CERT2 provides clinically meaningful additional information when incorporated into multivariable risk models. Its incremental utility should be evaluated through changes in discrimination, calibration, and risk reclassification after adjustment for established cardiovascular risk factors [51]. Therefore, while CERT2 may complement conventional cardiovascular risk assessment, further validation across independent and diverse populations is needed to determine whether its addition meaningfully improves clinical decision-making.

### 4.2. Ceramides in Statin-Treated/LDL-Optimized Patients

Cardiovascular disease is the leading cause of mortality worldwide, with roughly 70% of CVD event risk still seen in placebo-controlled statin trials for patients, despite treatment [52]. Here lies the preventative gap in solely cholesterol-focused risk panels, where ceramides, and specifically their long-chain to very-long-chain ratios, have been positioned as accelerators rather than bystanders to this residual risk, raising the question of whether statin treatment can truly treat or simply mask the ceramide-associated component of risk [7]. What worsens this issue is that LDL-C itself becomes a less reliable predictor in treated, older, and comorbid patients, in whom U-shaped or inverse associations with mortality are common [53]. Ceramide-based markers appear less vulnerable to this bias: ceramides improved risk prediction beyond LDL-C in a high-risk Austrian cohort [53] and outperformed LDL-C for cardiovascular mortality in a 16-year cohort in which about half of patients were taking statins [54]. CERT2 also retained prognostic value in hemodialysis patients enrolled in the rosuvastatin trials AURORA and 4D, although its discrimination was modest there [48].

Now, statins do lower circulating ceramides, but they are not a uniform intervention [55]. Ceramides travel largely on LDL particles, so LDL-lowering therapies can and do reduce them in part by removing their carriers [49,56]. Simvastatin lowered plasma ceramides broadly by about 25%, whereas ezetimibe, which also lowers LDL-C, produced no ceramide change, and a PCSK9 loss-of-function variant lowered risk-associated ceramides by about 20% [57]. Performed more recently, atorvastatin (40 mg) and simvastatin (80 mg) each lowered the CERT2 score by a median of 2 points on its 0–12 scale after eight weeks, while a PCSK9 antibody lowered it by 3 points [49]. For 292 patients with CAD, PCSK9-inhibitor use lowered Cer(d18:1/16:0), Cer(d18:1/18:0), and Cer(d18:1/24:0) by 22.9%, 24.5%, and 34.8% in comparison to statin therapy alone [58]. Here, the protective Cer(d18:1/24:0) fell the most, so the effect on ratio-based scores is not obvious from concentrations alone in these small or non-randomized studies. An important thing to notice is that studies did not show that lowering ceramides is what produces clinical benefit.

From these findings come two practical consequences: first, statin use is a real confounder since most high-risk cohorts are heavily treated; baseline ceramide concentrations and the thresholds derived from them reflect confounded, altered physiology: on-treatment reference ranges are still needed. Second, the responsiveness of ceramides to therapy raises the possibility of using repeating serial CERT2 measurements to gauge treatment response or adherence. The developers of CERT2 themselves stated that this requires evidence, ideally from a large randomized statin trial, and that outcome benefit is mediated by CERT2 reduction [49]. Until then, ceramide-based scores are best regarded as complementary to LDL-C, an additional informative measure of risk and concern.

These findings have important implications for interpreting CERT2 scores in patients receiving lipid-lowering therapy. Statins and PCSK9 inhibitors may reduce circulating ceramide concentrations and CERT2 scores, potentially influencing risk assessment in treated populations. However, treatment-related reductions in CERT2 scores do not necessarily indicate a corresponding reduction in cardiovascular events or improved clinical outcomes. Although serial CERT2 measurements may offer potential value for monitoring treatment response, their clinical utility remains unvalidated. Further prospective studies and randomized clinical trials are needed to determine whether changes in CERT2 scores independently predict improved cardiovascular outcomes [49]. Until then, CERT2 should be regarded as a complementary risk assessment tool rather than a validated surrogate marker of treatment efficacy.

### 4.3. Ceramides and Atherosclerosis

Atherosclerosis is the buildup of plaque deposits in arteries, interfering with blood flow and causing massive harm [59]. Ceramides are intertwined with the atherosclerosis mechanism, with inhibition of ceramide synthesis reducing palmitate-caused vascular and endothelial dysfunction, implicating ceramide synthesis as inherently atherosclerotic [59,60]. Ceramide activates PP2A to bind and dephosphorylate eNOS instead of I2PP2A, meaning that ceramide generation reduces nitric oxide, worsening vasodilation and increasing atherosclerotic risk [59,61]. Furthermore, ceramides are enriched in plaques, promoting LDL aggregation and retention, promoting atherosclerotic disease, and are elevated 10–50-fold higher near aggregated LDL than with plasma LDL and 2–3-fold higher in atherosclerotic aortic streaks in a 43-year-old heart donor [62]. This is because sphingomyelinase (SMase) hydrolyzes sphingomyelin, which is predominantly found in LDL shells, into ceramides and phosphorylcholines. The hydrophobic, hydrogen-bonding nature of ceramides means that LDL particles nearby must clump together and eventually lock towards the arterial intima due to extracellular proteoglycans [63]. These stuck LDL-ceramide groupings then become engulfed by macrophages, transforming the macrophages into foam cells that comprise atherosclerotic plaques [62]. Finally, the ceramide/S1P rheostat (Figure 3) also plays a part in the inflammation-endothelial response, with S1P signaling guarding against ceramide synthesis [64]. Serine palmitoyltransferase, the enzyme starting de novo sphingolipid synthesis, is inhibited by NOGO-B (reticulon-4B), an endoplasmic reticulum protein upregulated during hemodynamic stress in the endothelium around coronary lesions in humans and mice [64]. Yet mice lacking NOGO-B in their cardiac endothelial cells had resistance to coronary atherosclerotic mortality and progression, signifying that sphingolipid metabolism had tilted more toward S1P, which is more protective for the endothelial cells [64].

### 4.4. Sudden Cardiac Arrest and Death

Sudden cardiac arrest (SCA) is the rapid termination of heart movement and activity; sudden cardiac death (SCD) is death presumably occurring due to cardiac reasons within 1 h of onset of cardiological issues or within 24 h of the patient’s last healthy state [65,66]. SCA usually presents before SCD, and SCD is often the first indicator of cardiovascular disease [66]. The most common biomarker for abnormal substrate or structural heart disease is measuring Left Ventricular Ejection Fraction (LVEF), how much blood the left ventricle pumps out with each contraction; yet a majority of SCA deaths occur in individuals with normal or mild reductions in LVEF (above 35–40%); despite SCA’s involvement in over 300,000 deaths in Americans yearly, LVEF for risk stratification for SCA and SCD is still weak [67]. Furthermore, many of these high-risk, undetected individuals experiencing SCA have undiagnosed structural or cardiovascular issues, like CAD or CHD.

In a predominantly Caucasian cohort of *n* = 4612 with 215 SCD cases, after adjusted Cox proportional hazards regression analyses, Cer(16:0) and SM(16:0) were associated with significantly higher hazard ratios for SCD for higher SD of log sphingolipid levels, not altered by inclusion of sex, race, BMI, or baseline age [68]. Hazard ratios of 1.34 [95% CI, 1.12–1.59] for Cer(16:0) and for SM(16:0), 1.37 [95% CI, 1.12–1.67] implicates these lipids as potential areas for investigation of SCD. Further research could enable better detection of SCA and SCD events, a small yet severe subset of CVD events.

### 4.5. Hypertension

Hypertensive patients face increased residual risk despite treatment, making accurate prediction of MACE and mortality highly necessary; yet, their residual risk remains overlooked in current lipid panels [69,70]. Ceramides, but specifically long-chain ceramides, play an important role in endothelial dysfunction, as described in Section 4.3, the LacCer-eNOS interaction for SLE in Section 8.3, and the CKD-hypertension relation [71].

A study by Zhang et. al. applied CERT to a group of 562 male patients, then followed up for MACE after an average of 2.3 years. They found that Cer(d18:1/16:0), Cer(d18:1/22:0), and Cer(d18:1/24:0) provided significantly higher prediction results for MACE, in comparison to traditional variables like hypertension-involved organ problems, comorbidities, and common CV risk factors. The same study went on to create CERT-HBP, a ceramide-based risk scoring system for high-risk hypertensive patients for MACE, using Cer(d18:1/16:0), Cer(d18:1/24:1), alongside their ratios to Cer(d18:1/24:0) and Cer(d18:1/22:0) [70]. CERT-HBP significantly improved the C-statistic from 0.751 to 0.791, in comparison to CERT-1, which only insignificantly improved to 0.762 [70].

Following up on this study was a 10-year analysis of 7722 FINRISK participants, where ceramide and phosphatidylcholine ratios, specifically ceramide (d18:1/18:0), ceramide (d18:1/24:1), phosphatidylcholine (16:0/16:0), and ceramide (d18:1/18:0)/(d18:1/16:0), were associated with prevalent and new-onset hypertension, with both being associated with ceramide (d18:1/24:0) [69]. However, only CERT2 after covariate adjustment could predict new-onset hypertension; neither could significantly associate with prevalent hypertension [69]. As studies advance, applications of ceramides like CERT-HBP could help highlight high-risk hypertensive patients needing more directed therapeutics and treatment.

### 4.6. Heart Failure with Preserved Ejection Fraction

Heart failure with preserved ejection fraction (HFpEF) affects approximately 32 million people, almost half of all heart failure cases [72]. Unlike its reduced counterpart, HFpEF does not possess a single, consistent structural physical marker or lesion, so usual biomarkers like natriuretic peptides lack consistency, especially in affected obese and older female patients, complicating accurate diagnosis [72,73]. Through myocardial lipotoxicity and mitochondrial alterations mentioned in Section 3, insulin-resistance, inflammatory, and metabolic pathways from dysregulated ceramides combine to cause the diastolic stiffness found in HFpEF [72,74]. Furthermore, the liver–heart and gut–heart ceramide metabolic axes are involved, alongside Magaye et al. linking pregnancy, menopause, and aging in women to ceramide dysregulation, implicating ceramide panels as a plausible early or preventive marker in underdiagnosed, complex populations [72,73,75]. Further work with HFpEF-adjudicated human cohorts is needed, however, to make the jump to clinical validation.

### 4.7. Congenital Heart Disease

Congenital heart disease and its relation to ceramide and sphingolipid biology remains restricted to gene-expression and cellular-level studies rather than clinical-level work. For tetralogy of Fallot, metabolomics and gene-set enrichment have found general sphingolipid metabolism downregulation in chronically hypoxic environments, with reduced expression of ceramide-pathway genes such as UDP-glucose ceramide glucosyltransferase (UGCG) and sphingosine-1-phosphate phosphatases (SGPP1/2) [76]. While the significance of the downregulation of sphingolipid metabolism is not yet understood, further studies could potentially elucidate this relationship.

### 4.8. Sex Differences

Biological sex is another important source of variation in ceramide metabolism and may need to be considered when establishing clinical reference ranges. Estrogen signaling is mechanistically linked to sphingolipid homeostasis, and ceramides and S1P appear to be differentially regulated in males and females [77]. In a human cross-sectional study, plasma Cer(d18:1/24:1) was inversely correlated with circulating estradiol in women, but not in men, while complementary estrogen-receptor-positive cell experiments showed that estradiol exposure reduced ceramide accumulation [78]. These observations provide a biochemical rationale for why menopause and declining estrogen concentrations may alter the circulating ceramide profile and, consequently, the relationship between ceramide species and cardiovascular risk. Consistent with this possibility, researchers found that associations between plasma ceramides and arterial stiffness were particularly apparent in male participants, although their cross-sectional design does not establish estrogen as the causal mechanism [25]. Sex therefore should not be treated only as a statistical adjustment: it may reflect genuine hormone-dependent differences in sphingolipid metabolism [25]. This is clinically relevant because the extended CERT2 prognostic model incorporates age and sex alongside the lipid score [9]. Together, these findings support further development of sex-, age-, and potentially menopausal-status-specific ceramide reference intervals rather than assuming that a single concentration threshold will have equivalent biological meaning across populations.

### 4.9. Glycosylated Ceramides and CVD

Recent studies highlight glycosphingolipids as emerging mediators of myocardial injury and remodeling. Work in human myocardial biopsies and experimental models demonstrates that glycosylated ceramides (GlcCer) accumulate in dysfunctional or ischemic myocardium and can alter cardiomyocyte signaling. Andersson et al. demonstrated that loss of glucosylceramide synthase (GCS) disrupts Beta-1-adrenergic receptor trafficking, underscoring the dependence of adrenergic signaling on intact glycosphingolipid metabolism [79]. These findings point to glycosylated ceramides and sphingolipids as regulators of cardiomyocyte responsiveness rather than passive metabolic byproducts.

Ceramide metabolism also impacts severity of ischemia–reperfusion injury. Reforgiato et al. (2016) found that inhibiting de novo ceramide synthesis minimizes reperfusion injury by suppressing downstream apoptotic and inflammatory signaling pathways that include lactosylceramide (LacCer) [80]. This paper supports the model in which both ceramides and their glycosylated derivatives shape the cellular response to ischemic stress. Growing mechanistic evidence points to LacCer as a signaling lipid linking metabolic dysfunction to cardiovascular pathology [81,82]. LacCer activates inflammatory and oxidative pathways, including NADPH oxidase and MAPK signaling, that promote endothelial dysfunction and propagate tissue injury [83]. Furthermore, emerging translational data emphasize the direct, non-ischemic consequences of LacCer accumulation: Turner et al. reported that LacCer impairs cardiomyocyte contractility in hypoplastic left heart syndrome, expanding the relevance of glycosphingolipids beyond ischemic disease [84].

Collectively, these studies suggest that glycosylated ceramides such as GlcCer and LacCer may serve as mechanistic biomarkers of myocardial stress and dysfunction. Glycosylated ceramides’ consistent elevation across ischemic injury, cardiomyopathies, and inflammatory states raises the possibility that incorporating glycosphingolipid species into ceramide-based cardiovascular risk prediction frameworks could improve discrimination and enhance mechanistic interpretation of lipidomic profiles. While CERT and CERT2 tests currently do not include glycosylated ceramides, they have shown consistent association with myocardial stress and inflammation, which suggests they represent a potential gap in current frameworks. Ongoing efforts in lipidomics to expand its correlation in prospective diseases demonstrate a progression toward improving cardiovascular risk prediction.

### 4.10. Additional Joint CVD-Lipid Species and Interactions

The rise in understanding in lipidomics has broadened our understanding of cardiovascular diseases by highlighting that the risk of this disease cannot be solely explained by conventional lipids such as LDL, HDL cholesterol, and triglycerides. In addition to them, molecular lipid species including sphingomyelins, phosphatidylcholines and fatty acid species provide a deeper understanding of metabolic alteration, inflammation, and cardiovascular dysfunction.

Sphingomyelins (SMs) are widely distributed across plasma lipoproteins and arterial cell membranes, where they play necessary structural and signaling roles. SMs are the predominant lipids in the brain as well as in axons for neurotransmission [85]. Over the past decade, there has been growing understanding that certain SM species are not simply the components of lipid bilayers but actively reflect and contribute to cardiometabolic stress, inflammation, and destabilization of atherosclerotic plaques. Multiple cohort studies consistently demonstrate that higher circulating levels of SM 16:0 are associated with an increased risk of future cardiovascular events. For example, Chen et al. reported that palmitoyl sphingomyelin (PSM 16:0) was a significant predictor of incident cardiovascular disease in patients with type 2 diabetes [85]. Importantly, this association remained the same even after adjusting for conventional lipid markers including LDL cholesterol and triglycerides as well as established clinical risk scores, highlighting the independent prognostic value of certain SM species [85].

Phosphatidylcholines comprise the largest glycerophospholipid class in circulation, and their predictive role in CVD stems from both PC molecular species and from PC derived metabolites. Studies have observed direct associations of PC species and CVD risk, such as with lysoalkylphosphatidylcholine. In addition, several alkylphosphatidylcholine [PC(O)] species and alkenylphosphatidylcholine [PC(P)] species containing primarily saturated and monounsaturated fatty acids were directly associated. In contrast, the species containing polyunsaturated fatty acids like phosphatidylcholine and alkenylphosphatidylcholine were inversely associated with future cardiovascular events [86]. A major discovery came from Wang et al., who showed that certain metabolites of the dietary lipid phosphatidylcholine, mainly choline, trimethylamine N-oxide (TMAO), and betaine, predicted risk for CVD in an independent large clinical cohort [87]. Dietary PC serves as a substrate for gut microbiota to produce trimethylamine (TMA), later converted to TMAO (trimethylamine N-oxide), a metabolite linked to endothelial dysfunction, atherogenesis, and major adverse cardiovascular events. In their study, higher circulating TMAO predicted myocardial infarction, stroke, and death independent of traditional risk. This implicates certain PC species as markers of atherogenic microbial metabolism [87].

## 5. Ceramides and Cardio-Metabolic Diseases

Cardiovascular disease most often arises from prolonged metabolic dysfunction rather than isolated vascular pathology, and mounting evidence positions ceramides as lipid mediators that integrate insulin resistance, obesity, and dyslipidemia into downstream cardiovascular risk. Across population-based cohorts and mechanistic studies, ceramides consistently associate with metabolic diseases that substantially elevate CVD risk, including type 2 diabetes, obesity, and metabolic syndrome, and these associations persist even after adjustment for LDL cholesterol and traditional cardiometabolic risk factors [2,9,88].

### 5.1. Diabetes

Type 2 diabetes represents the most extensively studied metabolic condition linking ceramides to cardiovascular outcomes, and multiple prospective studies demonstrate that specific long-chain ceramides such as Cer(16:0) and Cer(18:0) predict both incident diabetes and future cardiovascular events. A large longitudinal study in *Diabetes* showed that elevated plasma ceramides and unfavorable long-chain to very-long-chain ceramide ratios predicted progression to type 2 diabetes independent of BMI, fasting glucose, LDL cholesterol, and triglycerides, suggesting that ceramides capture an early metabolic signal not reflected by conventional lipid measures [89]. Importantly, individuals with the highest ceramide risk profiles exhibited significantly greater cardiovascular mortality during follow-up, reinforcing the concept that ceramides mark shared metabolic pathways underlying both diabetes and cardiovascular disease [9,10].

Mechanistically, ceramides have been shown to impair insulin signaling through inhibition of Akt phosphorylation, disruption of mitochondrial fatty acid oxidation, and promotion of ectopic lipid accumulation in liver, skeletal muscle, and myocardium, and these effects directly contribute to insulin resistance and metabolic inflexibility. Experimental models demonstrate that saturated fatty acid-driven de novo ceramide synthesis induces lipotoxic stress, oxidative damage, and inflammatory signaling, all of which are established contributors to endothelial dysfunction and atherogenesis [18].

Obesity and metabolic syndrome further reinforce the role of ceramides as integrative markers of metabolic stress, and circulating ceramide profiles correlate strongly with visceral adiposity, systemic inflammation, and impaired glucose tolerance. Lipidomic analyses consistently demonstrate elevation of long-chain ceramides alongside depletion of protective very-long-chain species such as Cer(22:0) and Cer(24:0), resulting in adverse ceramide ratios that mirror those observed in high-risk cardiovascular cohorts [8,88]. This pattern reflects pathological ceramide remodeling and the notion that metabolic overload drives a shift toward pro-atherogenic ceramide species long before overt CVD becomes clinically apparent.

### 5.2. Non-Alcoholic Fatty Liver Disease

Increasingly, hepatic ceramide remodeling has been implicated in non-alcoholic fatty liver disease (NAFLD) and its progression to non-alcoholic steatohepatitis (NASH), now more commonly referred to as metabolic dysfunction-associated steatohepatitis (MASH) [90,91]. While total hepatic ceramide levels increase with the development of fatty liver disease, a marked shift in the acyl chains of hepatic ceramides occurs such that steatotic and MASH livers characteristically contain an increased proportion of short- to medium-chain ceramides, whereas very-long-chain ceramides, generated by CerS2, the predominant ceramide synthase in the liver, are considered to be of a protective nature in hepatic cells [90,91,92]. Short- to medium-chain ceramides, particularly Cer(16:0), synthesized by CerS5/6, are associated with various forms of lipotoxicity, including impairment of mitochondrial function, induction of endoplasmic reticulum stress, and elicitation of pro-inflammatory signaling pathways [90,92]. Thus, an imbalance between very-long-chain and short- to medium-chain ceramides, which can lead to hepatic triglyceride accumulation and to insulin resistance, may further exacerbate the cellular stress of simple steatosis, resulting in the transition to MASH [93]. Notably, the hepatic ceramide content is increased in patients with NAFLD/NASH, and reduction of hepatic ceramide synthesis has been shown to improve steatosis and to reduce features of inflammation in experimental models of fatty liver disease [92,93]. Thus, ceramide accumulation and its species-specific remodeling can influence NAFLD/NASH pathogenesis as active modulators of disease, rather than as mere liver injury markers.

The relationship between ceramides and cholesterol further highlights their complementary role in cardiovascular risk assessment. While LDL cholesterol remains causally linked to atherosclerosis and remains indispensable for therapeutic decision-making, its predictive power is diminished in metabolically heterogeneous populations such as individuals with diabetes or obesity [7,56]. Conceptual frameworks such as those laid out in Tippetts et. al. emphasize that residual cardiovascular risk persists despite optimal LDL lowering, and multiple studies demonstrate that ceramide-based scores retain prognostic value even in statin-treated populations with well-controlled cholesterol levels [7,9]. These findings suggest that ceramides capture lipid-driven cellular stress pathways that are orthogonal to circulating cholesterol burden.

## 6. COPD

Recent advancements have allowed lipidomic profiling to enable more insight into the metabolic constitution of patients with chronic obstructive pulmonary disease (COPD). Rather than dissecting a single pathway, these studies captured more global changes in metabolism, and one of the measures that is consistently disrupted is sphingolipid metabolism. In particular, Gharib et al. reported an inverse association with lung function in older CHS participants [94,95]. What is also interesting in this study is that these associations were identified in plasma, consistent with the idea that such lipid changes are not solely restricted to the lung and might mirror systemic events. Similarly, Wu et al. reported metabolic changes in patients with COPD, with lipid pathways, including sphingolipids, specifically affected [96].

The COPDGene cohort, a large study conducted with over 10,000 smokers and non-smokers to determine the underlying genetic traits, subtypes, and environmental factors of COPD progression variability in smokers, has provided some of the most systematic evidence linking sphingolipid metabolism to COPD phenotypes. In an early analysis of 211 COPDGene subjects, Bahr and colleagues found that peripheral blood mononuclear cell expression of sphingolipid pathway enzymes, together with plasma ceramide levels, distinguished COPD and emphysema status even after adjusting for smoking history, suggesting that ceramide dysregulation reflects disease processes beyond smoke exposure alone [97]. Furthermore, this study observed that lactosylceramide was present in all subjects with emphysema and some smoking control subjects, but not in patients with COPD and without emphysema. Building on this, Bowler and his colleagues conducted a targeted metabolomic analysis of 129 COPDGene subjects and identified five specific sphingomyelin species significantly associated with emphysema severity, along with a distinct subset of trihexosylceramide and dihexosylceramide species associated with COPD exacerbations [98], emphasizing that sphingolipid metabolic pathways are disease-specific and supporting a species-specific model for ceramide-based biomarkers.

An important follow-up by Siska and Bowler directly compared sphingolipid gene expression data with sphingolipid metabolite levels using a discordant approach [99], and found that fluctuations in metabolite levels could not be solely explained by gene expression [100], suggesting posttranslational regulation, altered enzymatic activity, or downstream activity as more likely drivers of the sphingolipid remodeling.

As a development on the species-specific issue, a large, population-based cohort study by Shoghli and colleagues provided the first test of whether the CERT1 and CERT2 cardiovascular risk scores, already established for CVD, also predict COPD [101]. Using data from the Finnish FINRISK 2002 cohort, the authors found that both CERT1 and CERT2 were significantly associated with prevalent and incident COPD in unadjusted models, though these associations were substantially reduced after accounting for cofactors such as age and smoking status, with only CERT2 remaining significant after this adjustment. Among individual lipid species and ratios, Cer(d18:1/14:1) emerged as the robust predictor of prevalent COPD, independent of smoking-related metabolic changes, while the Cer(d18:1/18:0)/PC(14:0/22:6) ratio was significantly associated with both prevalent and incident COPD across nearly all adjusted models, suggesting it may serve as a more reliable risk marker than either lipid class alone. Additionally, the study also found clinically meaningful sex differences: CERT2 remained a significant predictor of incident COPD in men after adjustment, but not in women, and there were markedly steeper declines in COPD-free survival among smokers, with comparatively little discriminative value among non-smokers [101]. The authors conclude by acknowledging the limitations of their observational design in implementing CERT scores on COPD, citing false negatives in mild cases and the inability to draw causal conclusions from observational data as limiting to their findings. However, taken with the previous evidence, this study further emphasizes that ceramides are shown to have greater risk marker or therapeutic potential in patients with COPD if the right species ratio can be identified.

Concurrent with this, there is increasing interest regarding the potential role of ceramides in structural and functional changes characteristic of COPD. Ceramides are involved in many processes, such as apoptosis and inflammation, that contribute to the pathogenesis of emphysema [102]. One relevant pathway may be that high levels of ceramide lead to alveolar cell destruction, resulting in a gradual loss of lung structure. More recently, Huang et al. provided additional insight by showing that macrophages secrete ceramide-rich extracellular vesicles. These vesicles appear to function as messengers for inflammatory signals and may also amplify tissue damage in the lung, providing a more dynamic understanding of COPD [102].

Additionally, a study conducted using an elastase-induced model of emphysema in mice found that a myrocin-mediated blocking of the de novo pathway of ceramide synthesis resulted in improved lung function after establishing a resultant increase in multiple ceramide species levels following elastase injection [14]. Complementing this is a study done by Arshad and colleagues, which compared plasma, phospholipids, ceramides and acid sphingomyelinase across patients with community-acquired pneumonia, infection-associated COPD exacerbation, and matched controls [103]. While lipid reprogramming was substantially more pronounced in pneumonia and COPD exacerbation than in healthy controls, both conditions showed increases in ASMase activity relative to controls, accurately distinguishing COPD exacerbation and pneumonia from healthy controls. Because ASMase catalyzes the breakdown of sphingomyelin into ceramides, this experiment provides evidence that multiple ceramide synthesis pathways are implicated in different COPD subtypes.

Taken together, all this evidence suggests that ceramides are not just passive end products of disease but could also be more actively related to its severity. However, despite these advances, there are some clear limitations that complicate what we can take away. Most human data, such as that generated by Gharib and Wu, are observational, making it difficult to determine whether altered lipid levels actually promote disease or merely reflect it [95,96]. Another problem is that plasma measurements do not necessarily reflect what is happening in lung tissue, where COPD pathology occurs. Rather, heterogeneities in study design and lipidomic methods also limit the comparability between cohorts. Finally, mechanistic studies such as those of Huang et al. provide valuable and even critical insights, but translating the findings into therapeutics is not straightforward, also due to the highly complex nature of lipid signaling pathways [102]. Additionally, the vast quantity of ceramide species that play an important role in different disease pathways of COPD makes it difficult to conclude which species, if any, could best serve as a risk marker or therapeutic target in COPD [14,99,100].

Potential confounding factors further limit interpretation of these associations. Age and smoking history are particularly important in COPD: the FINRISK analysis reported attenuation of CERT associations after adjustment for these variables, and sex-specific differences were also observed [101]. Renal function and concurrent medications may additionally influence circulating lipid profiles or differ between patient groups, but their effects have not been consistently evaluated in the studies summarized here. Future studies should report and adjust for age, sex, smoking exposure, renal function, and relevant treatments, and assess whether associations remain reproducible across COPD phenotypes and independent populations.

## 7. IBD

Inflammatory Bowel Disease (IBD) is a chronic condition characterized by sustained, recurrent inflammation of the gastrointestinal tract. It encompasses Crohn’s Disease (CD) and ulcerative colitis (UC) as its most important subtypes, and presents clinically with symptoms including weight loss, diarrhea, abdominal pain, and fatigue. These disease subtypes differ in their anatomical distribution and histopathological features; both arise through complex interactions among genetic susceptibility, immune dysregulation, epithelial barrier dysfunction, environmental risks, diet, and alterations within the gut microbiome. Increasing evidence links disturbances in sphingolipid metabolism with pathogenic processes. Specifically, ceramides, S1P, sphingomyelins, and glycosphingolipids serve as bioactive lipids that influence epithelial survival, microbial interactions, and intestinal barrier integrity [15]. Importantly, sphingolipid effects are context-dependent, and differing lipid species may exert opposing effects depending on their acyl-chain length, cellular source, subcellular localization, and metabolic conversion. Therefore, IBD appears to exhibit broad remodeling of the sphingolipid network, rather than a uniform rise or fall of ceramide abundance.

As previously discussed, clinical lipidomic studies increasingly support the potential utility of ceramide profiles as biomarkers of intestinal inflammation. Elger et al. measured eight ceramide and five hexosylceramide species in serum from patients with IBD, primary sclerosing cholangitis (PSC), combined PSC-IBD, and healthy controls. Patients with IBD had elevated serum levels of the long-chain species Cer(d18:1/16:0) and Cer (d18:1/18:0). Notably, the ratio of long-chain to very-long-chain (LC/VLC) ceramides was elevated in IBD and positively correlated with CRP and fecal calprotectin, two established measures of systemic and intestinal inflammation [104]. These findings indicate that the relative distribution of ceramide species may reflect inflammatory activity more effectively than total ceramide abundance alone.

The Elger study does address limitations that not all ceramide species change in response to IBD, and that between CD and UC, ceramide species were remarkably the same [104]. Ceramide species and levels were also associated with age, with a suggested hypothesis that sex and BMI had no impact on lipid analyses of IBD, though this was never tested [104]. The limited control and PSC study populations further warrant validation prior to clinical translation. Thus, the LC/VLC ratio serves as a candidate disease-activity marker rather than an established diagnostic tool.

Taken together, these findings suggest that elevated long-chain to very-long-chain (LC/VLC) ceramide ratios may serve as potential biomarkers of inflammatory activity in IBD. However, the available evidence remains preliminary, and further validation in larger, independent patient cohorts is necessary to establish their diagnostic accuracy, specificity, and clinical utility. Therefore, LC/VLC ceramide ratios should be considered investigational biomarkers rather than established diagnostic tools for IBD.

Fecal metabolomics reinforce that sphingolipid remodeling reflects intestinal disease activity. Among 302 patients with CD, Tan et al. used ultrahigh-performance liquid chromatography-tandem mass spectrometry to detect metabolites associated with disease activity, location, and phenotype. Active disease, characterized as fecal calprotectin greater than 100 μg/g, correlated with increased fecal ceramides, sphingomyelins, methylysine, and polyamines [105]. The combined metabolite model distinguished active from inactive CD, with an area under the receiver operating characteristic curve (AUROC) of 0.882. Additionally, metabolic patterns differed across inflammatory, stricturing, and penetrating disease phenotypes, as well as among colonic, ileal, and ileocolonic disease locations. These observations suggest that although sphingolipids mark inflammatory activity and present clinical diversity, the multi-metabolic nature of the model demonstrates that ceramide lacks specificity as a standalone biomarker.

Metabolomic analyses may also help predict therapeutic response. Ding and colleagues investigated serum, fecal, and urinary metabolic profiles in patients with CD starting anti-TNF therapy. Lipid profiling identified ceramides, sphingomyelins, phosphocholines, and triglycerides among the metabolites associated with a response or nonresponse, while bile-acid and amino-acid pathways also differed between groups [106]. Fecal lipid profiling exhibited particularly strong predictive performance for anti-TNF response, with a reported receiver operating characteristic value of approximately 0.94. These results indicate that sphingolipid alterations may have value not only for detecting active inflammation but also for identifying metabolic states associated with a treatment response. However, the small sample size and the multimetabolite nature indicate further validation in larger independent populations before clinical translation.

A mechanistic study involving murine small-intestinal organoids provides biological support for the associations observed in human lipidomic datasets. Within these organoids, Pauer et al. treated epithelial cells with TNF-α, interleukin-1β, and interferon-γ to model the inflammatory cytokine environment of IBD. Cytokine exposure altered chloride secretion and increased calcium-dependent signaling through changes between proteins IP3R1 and STIM2. The exposure also increased Caspase-3-positive cells and generated an “apoptotic lipidome” associated with elevated ceramides, sphingomyelins, and ether-linked lipids [107]. Imaging mass spectrometry localized ceramide-positive apoptotic cells within the organoid lumen. This study directly links proinflammatory cytokine exposure to altered calcium signaling, epithelial apoptosis, and sphingolipid accumulation, reinforcing that ceramide remodeling promotes and stems from epithelial injury during intestinal inflammation.

The ceramide-to-S1P ratio is crucial for maintaining intestinal immune homeostasis (Figure 3), as reported by Ma et al., who observed that expression of the RNA demethylase FTO (fat mass and obesity-associated protein) was reduced in intestinal epithelial cells from patients with UC. FTO deficiency increased methylation and reduced the stability of CerS6 mRNA, promoting lower CerS6 expression and increased S1P accumulation in epithelial cells [108]. Secreted S1P stimulated macrophages to produce serum amyloid A proteins, which induced Th17-cell differentiation and aggravated experimental colitis (Figure 3). This mechanism reveals why ceramide abundance cannot be interpreted in isolation. Reduced synthesis of one ceramide pool may redirect metabolic flux toward S1P, a potent proinflammatory and pro-survival lipid signal. Consequently, the biological effects are dictated by the dynamic equilibrium among ceramide turnover pathways and S1P signaling rather than on the concentration of an isolated metabolite.

Collectively, the literature supports a model in which IBD is characterized by broad remodeling of sphingolipid metabolism rather than inducing a predictable change in total ceramide abundance. Long-chain ceramide enrichment and elevated LC/VLC ratios appear to reflect inflammatory burden, while fecal sphingolipid profiles may provide additional information about disease activity, phenotype, and response to anti-TNF therapy. Mechanistic studies further demonstrate that ceramide, S1P, and glycosphingolipid pathways regulate epithelial apoptosis, calcium signaling, macrophage activation, Th17 differentiation, regulatory T-cell function, and host-microbiome interactions. However, no individual ceramide species or ratio has yet been sufficiently validated for routine clinical use. Larger multicenter longitudinal studies are needed to evaluate whether integrating species-specific lipid profiles with routine biomarkers (CRP and fecal calprotectin), endoscopic findings, and diagnostic metrics can improve disease monitoring, risk stratification, and therapeutic outcome prediction.

## 8. Autoimmune Rheumatic Diseases (AIRDs)

### 8.1. The AIRDs–Ceramide Interaction

Autoimmune rheumatic diseases (AIRDs) are disorders where the immune system attacks the body, with similar mechanisms and antibody response cascades, causing worsened mortality and morbidity for the patient. Within this set of diseases are rheumatoid arthritis, systemic lupus erythematosus, Sjögren’s syndrome, systemic sclerosis, psoriatic arthritis and a plethora of other disorders. Treatment for AIRDs generally focuses on addressing inflammation and antibody attack and improving remission chances. One major issue underlying AIRD therapies is their low target specificity, accelerating lipid and metabolic dysfunction alongside worsened cardiovascular health [11]. Furthermore, patients suffering from AIRDs have worse detection of their heightened cardiovascular risk. For example, rheumatoid arthritis can cause the “cholesterol paradox”, where patients have reduced total and low-density lipoprotein (LDL) cholesterol. Yet, these patients are actually at a higher risk of CVD, causing the said paradox [109]. As AIRD patients carry elevated CVD risk that conventional lipid markers consistently underestimate, ceramide-based biomarkers become attractive for appropriately stratifying CVD risk levels [11,109,110,111].

Ceramides are implicated in inflammation through several different pathways, many of which sometimes intersect, as they serve as signaling molecules for apoptosis, alter immune response, worsen endothelial dysfunction, and regulate production of other similar molecules like S1P and C1P [112]. With inflammation stemming from AIRD pathology, lipid metabolism’s severe alterations correspond with several studies showing that the concentration and variety of ceramides in human tissues are altered in patients with rheumatic diseases compared to controls. Ceramides are also associated with the development and presentation of a plethora of AIRD comorbidities, including dyslipidemia, increased CVD risk, and fibrosis [11].

While AIRD therapies have centered on suppressing inflammatory responses, this therapeutic convention possesses low target specificity, creating less predictable cell metabolic effects: one key example being lipid metabolism, where current therapies can improve or exacerbate dyslipidemia [110].

Neutrophils, a type of immune cell, are known to contribute to inflammation in joints through small membrane-bound particles called extracellular vesicles (EVs). When a joint becomes inflamed, neutrophils can accumulate in the synovial fluid. A study has been conducted in which the lipidome of SF-EVs in inflamed joints, derived from patients with RA and spondyloarthritis, was compared to the lipid profile of equine SF-EVs obtained in reduced acute synovitis [113]. Neutrophil stimulation intensity-dependent changes were identified in the lipidomic profile of nEVs with an elevated presence of lactosylceramide and other phospholipids [113]. The study concluded that lipidome profiling can help deepen our understanding of AIRDs by providing us with insights into the contribution of nEVs to the pool of SF-EVs.

Interpretation of ceramide associations across autoimmune rheumatic diseases also requires careful consideration of confounding. Age, sex, smoking, renal function, and concurrent medications may vary across patient populations and could influence measured lipid profiles independently of autoimmune disease activity. Although the RA cohort analysis adjusted for age, smoking, and several cardiometabolic and inflammatory variables [112], such adjustment is not necessarily consistent across studies or disease subtypes. In particular, renal involvement and differences in immunomodulatory or lipid-lowering treatment warrant consideration when comparing cohorts. Future investigations should document these factors, use appropriately adjusted analyses, and validate findings across diverse patient populations before assigning diagnostic significance to ceramide profiles.

### 8.2. Rheumatoid Arthritis

Rheumatoid Arthritis (RA) is a chronic condition that causes pain, swelling, and inflammation in the joints, characterized by hypoxic joints, occurring when the immune system mistakenly attacks its own body’s tissues, making it an autoimmune condition [114,115]. Patients with RA have a markedly greater likelihood of cardiovascular events, so the ceramide connection to RA has been directly examined using the CERT framework [114,116,117]. A 2025 FINRISK population-based cohort study by Shoghli and colleagues demonstrated a significant association between prevalent RA and the bioactive lipid species used for cardiovascular risk assessment [112]. CERT scores and their ceramide components were significantly associated with the prevalence of RA in both univariable and multivariable logistic regression analyses adjusted for age, HDL cholesterol, LDL cholesterol, BMI, smoking, and high-sensitivity CRP [112]. What stands out as important is that CERT scores were associated with prevalent RA but not with incident RA, implicating ceramide dysregulation as a reflection of the active disease phenotype rather than predicting disease onset.

Through lipidomics performed on synovial fluid and circulating ceramides, the potential for ceramide as a biomarker for RA has expanded. A metabolomic and lipidomic analysis of sphingolipid metabolism occurring in RA synovial fibroblasts in a hypoxic environment revealed 11 distinct sphingolipid metabolites relating to RA [114]. Generally, hypoxic joint intracellular environments seen in RA cause lactate accumulation and microenvironment acidity; this environment furthers A-SMase activity, worsening the inflammatory response [103,118,119]. But by treating MH7A cells with geniposide (GE), a common East Asian remedy for RA, changes in certain lipids were observed from their pre-treatment inflammatory hypoxic level. The levels of Arabia cerebroside, palmitoyl sphingomyelin, lysosmine (d18:0), SM (d18:1/18:0), Cer (d18:0/14:0) and Cer(d18:0/16:0) were increased in the hypoxic-RA environment, alongside Galactosylceramide (d18:1/20:0) being reduced [114]. But after 50 µM GE administration, all of these lipids’ levels returned to normal levels (except Galactosylceramide, which showed no change), pointing to ceramide metabolism and inflammation interplay being crucial in RA progression [114]. In further targeting of the SM/ceramide axis with precise lipidomics, associations between altered SM/ceramide metabolism and certain ceramides’ circulating level increases were demonstrated in a cohort of patients with progressing RA [111]. Specifically, eight ceramides were positively correlated with increased RA disease activity: SM (d18:0/18:2), SM (d18:1/18:1), SM (d22:2/20:1), SM (d24:0/18:2), Cer (d18:1/16:0), Cer (d18:1/18:1), CerP (d18:1/18:1), and CerP (d18:1/20:0), with seronegative and seropositive patients not having a significant difference in ceramide levels [111]. Furthermore, sphingomyelin phosphodiesterase 3 (SMPD3) was correlated with Cer (d18:0/16:0), Cer (d18:1/16:0), Cer (d18:1/18:0), Cer (d18:1/20:0), and Cer (d18:1/22:0), pointing to its role in regulating the SM circulation cycle but also promoting RA by inflammatory ceramide growth [111].

Consider that serum antibody-negative RA patients routinely fail detection of rheumatoid factor and anti-CCP through standard RA diagnostics: because seronegative RA is the most difficult form to diagnose, whether ceramide ratios could complement existing antibody tests deserves formal evaluation; to date, no study has assessed the diagnostic accuracy of ceramides for seronegative RA against an accepted reference standard [111].

Many in vitro and some in vivo animal studies have further researched the potential use of ceramides as therapeutic targets in RA, and since the majority of ceramide synthesis is diet-centric, dietary interventions may alter ceramide concentrations in the blood and affect health [11], which is a therapeutic avenue that deserves investigation. In tandem, acid ceramidase inhibition has been shown to regulate MHC class II antigen presentation and suppress autoimmune arthritis in preclinical models [120]. But while RA is the AIRD with the most developed link of ceramide-CVD risk, lupus contains the most thorough ceramide disease activity marker data.

### 8.3. Systemic Lupus Erythematosus

Lupus is a chronic autoimmune condition with no clear diagnostic criterion. While patients have a genetic predisposition, it is not a predictive prognosis and is often developed from a combination of environmental triggers. Systemic lupus erythematosus (SLE) is the most common form, and it is a heterogeneous disease characterized by the production of abnormal autoantibodies and immune complexes that result in widespread inflammation of organs and tissues. Most notably, the cardiovascular system is affected, which significantly increases the risk of CVD being the leading cause of death for SLE patients [121].

Emerging studies have suggested that the key effector cells involved in lipid metabolism dysregulation are modulators of the pathogenesis of SLE. Thus, imbalances in lipid homeostasis have been shown to contribute to SLE, with an association with the production and deposition of complement-fixing immune complexes and abnormal autoantibodies.

While it is established that ceramide and sphingolipid species are dysregulated in SLE activity, LacCer, the glycosphingolipid formed downstream of ceramide via the enzyme LacCer synthase, offers a much more direct mechanistic link to the vascular pathology underlying SLE-associated CVD. LacCer is generated when factors for atherogenesis, like oxidized LDL, TNF-α, and VEGF, upregulate β-1,4 galactosyltransferase. The resulting LacCer then promotes NADPH oxidase to generate ROS, creating nascent oxidative stress triggering inflammatory and atherosclerotic signaling [59,60,122,123]. This places LacCer mechanistically downstream of the ceramide elevations discussed, converting a correlational lipidomic signature into a proposed causal pathway toward vascular injury. LacCer has been shown to cause endothelial dysfunction in coronary arteries and endothelial cells through downregulation of eNOS and NADPH oxidase-driven oxidative stress, which is directly relevant given that cardiovascular involvement is the leading cause of SLE patient mortality described above [59,60,83,122,123]. LacCer also contributes to inflammation via cell adhesion/migration and phospholipase A2 pathways implicated in atherosclerosis; it also upregulates neutrophil Mac-1 expression, promoting leukocyte-endothelial cell adhesion, a mechanism that could connect lipid dysregulation to the immune complex deposition and vascular inflammation characteristic of SLE pathogenesis [123]. Despite this established role in atherosclerotic and endothelial pathology more broadly, LacCer itself has not yet been directly measured or localized within cardiac tissue in the SLE literature reviewed above, representing the specific gap this study addresses.

Dyslipidemia or the disruption in lipid metabolism refers to an increase in serum levels of LDL, TG, and TC. In population-based cohort studies, ischemic heart disease has been 10-fold higher in SLE patients in comparison to the general population. Additionally, children with SLE were found to be more susceptible to heart failure and dyslipidemia. The patients had a particular increase in ApoB and ApoA-1, which all contribute to atherosclerotic risk [12].

A study of 107 female patients with SLE identified a significant association between sphingolipids and SLE. Plasma levels of ceramide species, including Cer(18:0), Cer(16:0), and Cer(24:2), were significantly elevated in SLE patients compared to controls. Notably, the ratio of Cer(16:0)/S1P was demonstrated to be the best discriminator of SLE patients from healthy individuals, suggesting that sphingolipid ratios may serve as markers (Figure 3). The ratio correlated with Systemic Lupus Activity Measurement (SLAM) and Systemic Lupus Erythematosus Disease Activity Index (SLEDAI), which are measures that reflect current disease activity across different organ systems. Furthermore, immunosuppressive therapy normalized the abnormal sphingolipid levels, indicating that sphingolipids are dynamic and responsive to treatment. This suggests potential utility as markers that discriminate SLE from health and track treatment response, although these cross-sectional findings still require validation in independent cohorts [13].

Another prospective longitudinal study showed an increase in Cer(d18:1/16:0) and a decrease in longer-chain 20–22 carbons containing species (especially Cer(20:0)) in lupus patients compared to their population-matched controls. They were also indicated to be associated with an inflammatory response, as phosphatidylserines, known to be inflammation suppressors, were reduced in patients with lupus [124].

A study of 38 patients diagnosed with SLE included 27 without lupus nephritis (LN), 11 with biopsy-confirmed LN, and 30 healthy controls [125]. The lipid profile analysis showed a trend toward higher total and LDL cholesterol levels in the control groups. However, these differences were not statistically significant. When the sphingolipid profile was performed in patients with SLE (with and without LN) and healthy controls, the Cer16/S1P ratio was significantly higher in the SLE with LN group compared to the other two groups (0.33 vs. 0.27 vs. 0.25) [125]. In this exploratory pilot study, Cer(16:0)/S1P was the best-performing serum sphingolipid marker for discriminating LN, a finding that needs confirmation in larger cohorts [125]. A statistically significant positive correlation was observed between Cer(16:0)/S1P and disease duration, suggesting altered sphingolipid metabolism may contribute to SLE’s pathophysiology.

## 9. Chronic Kidney Disease/End-Stage Kidney Disease

Chronic kidney disease (CKD) has a close association with CVD; one of the most common reasons for morbidity and mortality in CKD patients is CVD, with increasing rates due to aging populations alongside increases in diabetes mellitus and hypertension incidence [48]. CVD can progress into ESKD, its final stage, requiring life-saving treatments like dialysis or organ transplantation [48]. Furthermore, CKD/ESKD patients, especially those undergoing hemodialysis, suffer from reverse lipid epidemiology, meaning that they display lower cholesterol levels despite an increased number of cardiovascular events [48,126]. Here, LDL-C is notoriously a poor marker of CVD risk, making ceramides valuable.

Cer(d18:1/16:0) and Cer(d18:1/18:0) were highlighted as elevated in CKD patients compared to non-CKD patients, even though both groups had similar CVD burden, with ceramides [127]. Lower eGFR correlated with higher concentrations of these two ceramides but also increased CVD risk [127].

**Figure 4:**
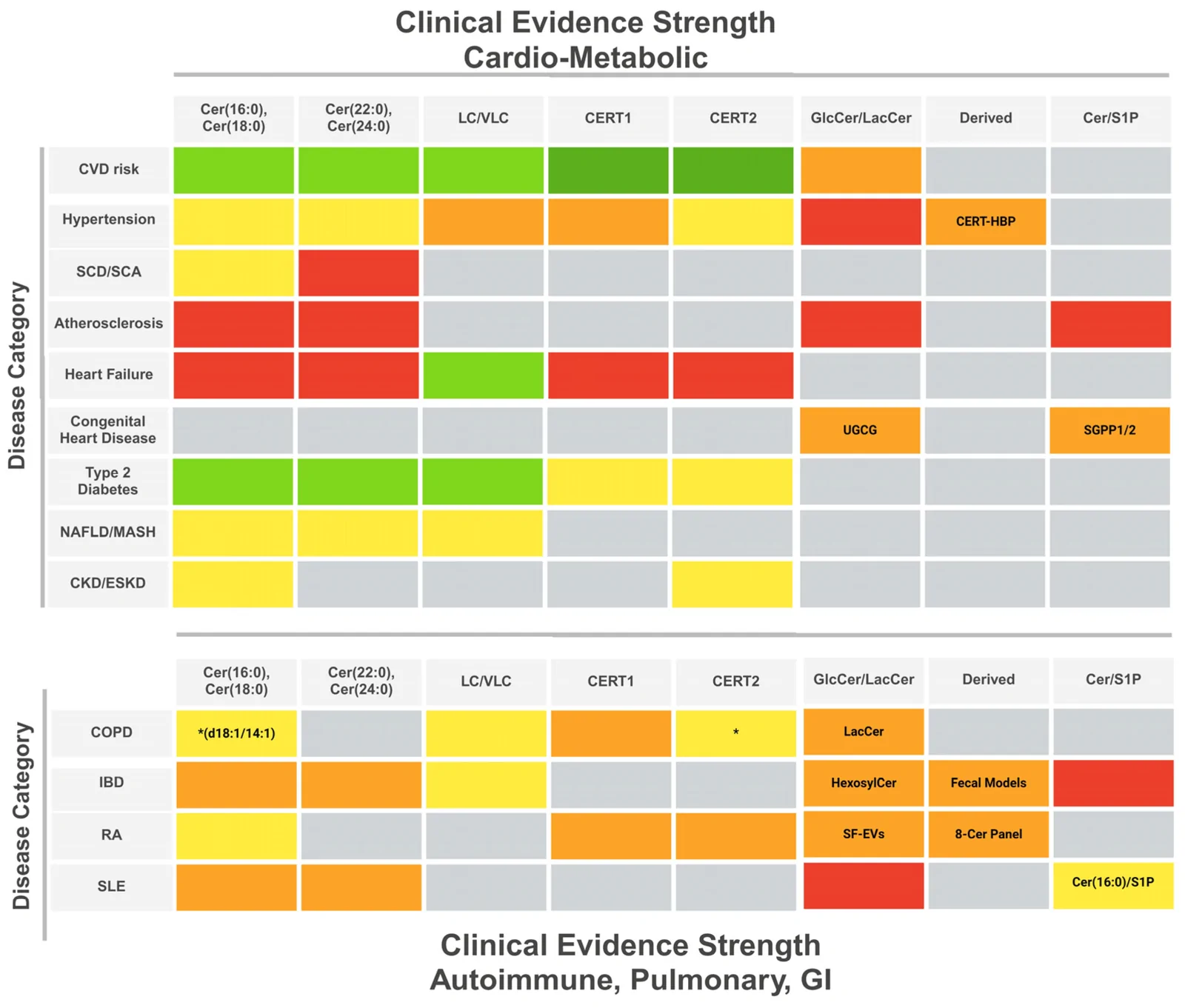
Strength of clinical evidence for ceramide species, ratios and scores in cardiometabolic and chronic kidney disease (top) and in autoimmune rheumatic, pulmonary (COPD) and gastrointestinal (IBD) disease (bottom). Colors indicate translational readiness as defined in Section 2.4: dark green, clinically deployed; light green, replicated in ≥2 independent human cohorts; yellow, single-cohort human evidence; orange, preliminary human evidence (small cross-sectional or case-control, tissue-only, not significant after adjustment, or scores not tested beyond their derivation cohort); red, mechanistic or preclinical evidence only; gray, no studies identified. Text within a cell names the specific marker or panel. Categories reflect replication and clinical application, not a formal assessment of study quality. * Association significant in men only (Shoghli et al. [101]). LC/VLC, long-chain to very-long-chain ceramide ratio; UGCG, UDP-glucose ceramide glucosyltransferase; SGPP1/2, sphingosine-1-phosphate phosphatases 1 and 2; SF-EVs, synovial-fluid extracellular vesicles. Created in BioRender.com.

Analysis of the A Study to Evaluate the Use of Rosuvastatin in Subjects on Regular Hemodialysis: An Assessment of Survival and Cardiovascular Events (AURORA) and Die Deutsche Diabetes Dialyze Studie (4D) trials reveals that the CERT2 score provided small but statistically significant predictive capabilities for CV death, all-cause death, and MACE (with c-indices 0.593, 0.595, and 0.581, respectively) [48]. Outperforming LDL-C in predicting these endpoints, CERT2 also improved SCORE2 and ASCVD predictive results, and CERT2 had the highest independent hazard ratios for the highest risk group [48]. Figure 4 summarizes the strength of the clinical evidence for the cardiometabolic, pulmonary, gastrointestinal, and autoimmune conditions discussed in Section 4, Section 5, Section 6, Section 7, Section 8 and Section 9.

## 10. Ceramides in Cancer

**Figure 5:**
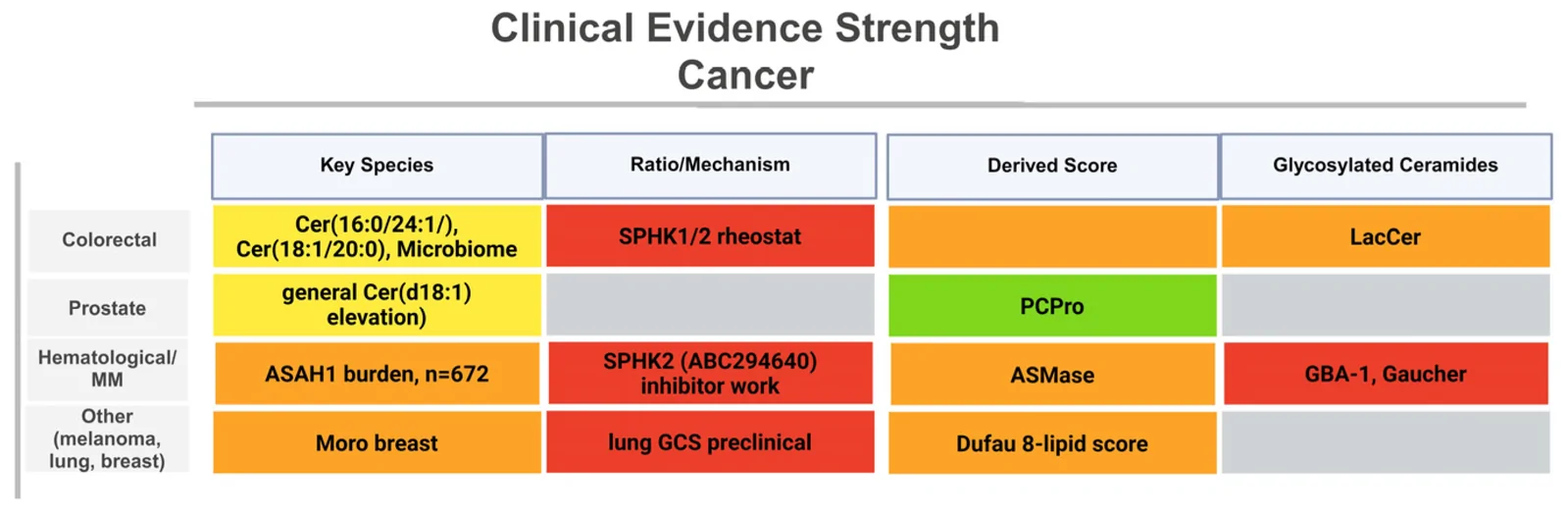
Strength of clinical evidence for ceramide species, mechanisms, derived scores and glycosylated ceramides in the cancers discussed in Section 10. Colors indicate translational readiness as defined in Section 2.4 (light green, replicated in ≥2 independent human cohorts; yellow, single-cohort human evidence; orange, preliminary human evidence; red, mechanistic or preclinical evidence only; gray, no studies identified); no cancer marker is clinically deployed. Mechanistic findings are shown in red even where they are extensive, because they do not establish biomarker utility in patients. ASAH1, acid ceramidase; ASMase, acid sphingomyelinase; GBA1, glucocerebrosidase; GCS, glucosylceramide synthase; MM, multiple myeloma; SPHK, sphingosine kinase. Created in BioRender.com.

Compared with the cardiovascular literature, most ceramide research in cancer focuses on tumor biology and therapy rather than on circulating biomarkers, so we separate three types of evidence throughout this section. Mechanistic evidence from cell lines, organoids, and animal models shows how ceramide metabolism shapes apoptosis, drug resistance, and tumor progression, and points toward therapeutic targets; on its own, however, it does not show that any ceramide measurement is useful in patients. Tissue-based evidence, comparing ceramide content or enzyme expression between tumor and adjacent normal tissue, supports biological relevance in human tumors but is invasive and usually cross-sectional. Clinical biomarker evidence requires circulating ceramide measurements in patients that relate to diagnosis, stage, survival, or treatment response, ideally adjusted for established clinical factors and validated in an independent cohort. Only this last type speaks directly to the question this review asks, and as Figure 5 shows, it remains limited: among the cancers discussed here, only the prostate cancer lipid signature PCPro has been evaluated in more than one independent patient population.

### 10.1. Cancer and Ceramide Mechanisms

Ceramides have been identified as significant mediators of tumor progression and cell death due to their position in apoptosis, stress signaling, angiogenesis, and responses to therapeutics. Extensive research has shown that cytotoxic chemotherapy and radiation therapy elicit ceramide accumulation, which is derived from both de novo synthesis and sphingomyelin hydrolysis, and that dynamic changes in ceramide levels are responsible for treatment-dependent apoptosis [128,129], specifically implicating Cer(d18:1/16:0) and Cer(d18:1/18:0) as pro-apoptotic drivers [40,130].

Resistance to cancer therapeutics is improved when tumor cells inhibit ceramide production or increase its catabolism, e.g., GCS, ceramidases, and sphingosine kinases (SPHKs), leading to a depletion of pro-apoptotic ceramide levels in the tumor and embarking it on a path to long-term survival [131,132]. Effectively, ceramide glycosylation allows tumor cells to avoid programmed cell death; Liu et al. demonstrated this in 2001 by transfecting adriamycin-resistant breast cancer cells with GCS, improving resistance to a variety of drugs (anthracyclines, Vinca alkaloids, taxanes, and other anticancer drugs) [42]. Increased GCS expression has thus been linked with multidrug resistance, metastasis, and poor clinical outcomes in breast, bladder, and colorectal cancer; inhibition of GCS can reverse chemosensitivity and mediate ceramide-dependent cell death [42,131,132]. Acid ceramidase (AC) is another metabolic-ceramide enzyme that, when overexpressed in primary prostate cancer cells and melanoma, appears to confer resistance to common cancer therapeutics, like doxorubicin, cisplatin, etoposide, gemcitabine or C6-ceramide [43,133].

Ceramides like Cer(d18:1/16:0) and Cer(d18:1/18:0) shape the tumor microenvironment through their interaction with S1P, forming a theoretical sphingolipid rheostat (Figure 3) [134,135]. Once ceramide decreases and S1P increases, tumors tilt towards pro-survival signaling, angiogenesis, immune suppression, and metastasis, characteristic of aggressive cancers (Figure 3) [131,134]. Thus, inhibition of ceramide-depleting enzymes (e.g., ceramidases; sphingosine kinases) can reverse apoptosis resistance and sensitize cells to chemotherapy and radiotherapy [41,128,130,131,132,134,136].

### 10.2. Breast Cancer

Breast cancer was the first cancer type with a clinical identification of ceramide dysregulation [137,138]. Moro et al. later demonstrated that while tumor tissues from the breast had higher ceramide content than normal breast tissue, higher levels of ceramide corresponded to less aggressive and tumorigenic cancers [137]. Furthermore, higher expression of ceramide-pathway related genes indicated worse prognosis for patients, which, together with the mechanistic GCS work below, suggests that dysregulated ceramide metabolism contributes to tumor progression and therapy resistance [42,137,139]. These are tissue-based findings, however, and none of the studies discussed here evaluated circulating ceramides as diagnostic or prognostic biomarkers in breast cancer. This supports Liu et. al’s example of GCS multidrug resistance with breast carcinoma cells, specifically vinblastine-resistant epithelioid carcinoma cells, emphasizing the linkage in the initial findings on cancer-ceramide resistance with breast cancer [42].

### 10.3. Colorectal Cancer

Colorectal cancer (CRC) has had a plethora of sphingolipid biology research, with investigation of ceramide dysregulation, metabolism, microbiology, and therapeutics [140,141]. Considering that CRC is pathologically heterogeneous, lipid profiles significantly differ based on tumor location (proximal vs. distal, rectal vs. colonic), metastatic stage, and mutational burden and background; that heterogeneity provides potential for ceramides as stratification markers and therapeutic targets [142,143].

Ceramide alterations point to epidemiological and lipidomic CRC detection at the tissue and plasma level; Markowski and his group found in 2020, through sphingolipid profile analysis of blood alongside healthy and tumor colorectal tissue from CRC patients using LC-MS/MS, significantly different ceramide content between CRC and surrounding healthy tissue [144]. Continually, they highlighted a relationship between tumor tissue ceramide concentration and plasma tracking TNM disease stage; ROC curve analysis of plasma’s C16:0, C24:1, C18:1, and C20:0 ceramides distinguished patients with advanced CRC (TNM III+IV) from earlier stage disease [144]. This was one of the earliest examples in solid tumor oncology that circulating ceramide species may reflect disease burden, although this single-center study of 45 patients reported only modest discrimination (AUC approximately 0.71–0.73) and its cutoffs have not yet been tested in an independent cohort [144].

The same group, through ultra-high-performance liquid chromatography alongside triple quadrupole mass spectrometry, demonstrated significant sphingosine and ceramide content differences between colonic and rectal cancers and their healthy tissue counterparts [29]. For rectal cancer, sphingosine content was elevated while C18:0 and C20:0 ceramides were lower than adjacent healthy tissue; colon cancer showed raised sphingosine, sphinganine, S1P, and C24:0 ceramide contents, implicating anatomical subtype as key for development of any CRC ceramide biomarker framework [29]. Lipidomics for staging was also explored with machine learning in an Australian cohort, where lipid panels discriminated across disease stages when combined with existing clinical CRC-pertinent variables; specifically, combining multi-omics features like IL-8, PF-4, MLH-1, alongside specific plasma PS and PE lipids is helpful for tumor progression in the early CRC stages [145]. This was an independent exploratory analysis with a different lipid panel, rather than a validation of the ceramide findings above.

The genetics driving ceramide alteration in CRC patients is partially understood through CerS isoform-specific downregulation linked to oncogenic mutations such as KRAS. Through enrolling and measuring 82 consecutive I-IV CRC patients’ sphingolipid metabolic gene expression and mutation profiles, CERS4 expression was significantly lower in the KRAS-mutant CRC subgroup in comparison to the KRAS-wild-type subgroup, with *p* = 0.004; CerS4 also predominantly generates C18:0 and C20:0, which were comparatively lower in the previous lipid profiling data [29,30]. Thus, KRAS-driven CerS4 downregulation posits a mechanism for oncogenic signaling to minimize pro-apoptotic ceramide pools [30]. Paradoxically, vascular invasion was also increased in CerS4 high-expression patients, implicating CerS4’s role in invasion-related pathways independent of its ceramide-generating function [30]. As tissue gene-expression findings from 82 patients, these results describe tumor biology rather than establishing CERS4 or C18:0/C20:0 ceramides as clinical biomarkers.

The gut microbiome was recently investigated for involvement in CerS activity and subsequent tumor progression [28]. Alongside GCS upregulation, which has also been identified in colorectal cancer specifically, contributing to chemoresistance, metabolic alteration was linked to CRC progression unexpectedly through the gut microbiome [28,131,132]. Primarily, Cer(d18:1/26:0) levels were elevated in CRC patients and mouse models—the ultra-long-chain Cer26:0 activated epidermal growth factor receptor (EGFR) by binding its extracellular region, promoting cancer proliferation [28]. Yet, the rise in C26 was driven by excess CerS3 activity, which was partially explained by Bacteroides cellulosilyticus, which produces riboflavin that inhibits CerS3 activity and thereby delays CRC progression [28].

The IBD-CRC transition also involves the ceramide/S1P rheostat mentioned prior; Espinoza and Snider emphasized that continued prevalence of S1P caused by elevated SPHK1 promotes CRC development through suppression of ceramide-mediated apoptosis [108,146]. SPHK possesses two isoenzymes, SPHK1 and SPHK2 [147]. For the first isoenzyme, elevated SPHK1/S1P activity in CRC cells was linked to increased pro-angiogenic function, earlier metastasis, and poorer clinical outcomes through the MIF-CD74/CD44 axis, driving M2-like macrophage polarization in the tumor microenvironment [148]. siRNA silencing of SPHK1 elevated ceramides and reduced intracellular S1P, with no impact on SPHK2’s mRNA and protein levels [147]. Yet, SPHK2 ablation elevated SPHK1’s mRNA, protein, and activity levels while heightening intracellular S1P [147]. Importantly, SPHK2-selective ablation reduced cell proliferation and invasion more than its SPHK1 counterpart [147]. For targeted inhibition of SPHK2, ABC294640 significantly inhibited CRC cell growth in vitro and in vivo when administered to xenograft nude mouse models; alongside S1P depletion and increased ceramide, the CRC cells were more sensitive to 5-FU and cisplatin [149]. Regardless of isoform, by promoting pro-survival immune evasion and growth in the colonic epithelium in preclinical models, S1P and SPHK remain promising therapeutic targets rather than established biomarkers [146].

### 10.4. Prostate Cancer

Prostate cancer has seen the most advanced ceramide-based biomarker advancements regarding prognosis in metastatic disease. PCPro, a lipid assay stratifying for overall survival in prostate cancer patients, measures Cer(d18:1/18:0), Cer(d18:1/24:0), Cer(d18:1/24:1), and Cer(d18:1/24:0)/Cer(d18:1/24:1) combined with total cholesterol and triglycerides [46]; there is better understanding of how ceramides can impact prognosis and survival for mCRPC [46,150]. It was developed in men with metastatic castration-resistant prostate cancer (mCRPC): in a discovery cohort of 105 men starting taxane chemotherapy, PCPro-positive men had shorter overall survival (OS) (HR 3.75), and this was confirmed in an independent validation cohort of 183 men with mCRPC starting taxanes or androgen receptor signaling inhibitors (HR 2.13) [46].

PCPro was subsequently evaluated in a different clinical state, metastatic hormone-sensitive prostate cancer (mHSPC), in a post hoc analysis of the phase III ENZAMET trial, which randomized men with mHSPC to androgen deprivation therapy (ADT) plus enzalutamide or ADT plus a conventional non-steroidal antiandrogen [150]. Enzalutamide is an androgen receptor (AR) inhibitor that blocks androgen binding to the AR, AR nuclear translocation and AR binding to DNA; it does not lower testosterone, which is suppressed by the concomitant ADT. In ENZAMET, PCPro-positive men (about 13% of the 866 with available samples) had shorter OS (HR 1.81), and the OS benefit of enzalutamide was seen in PCPro-negative but not PCPro-positive men [150]. These findings suggest that PCPro identifies a poor-prognosis subgroup in both mCRPC and mHSPC and may mark resistance to AR-targeted therapy, but, as a post hoc analysis, they require prospective confirmation before PCPro could guide treatment selection.

Furthermore, untargeted lipidomics in 48 patients with mCRPC showed that higher plasma Cer(d18:1/18:0) was associated with shorter overall survival after adjustment for prostate-specific antigen (PSA) and line of treatment, with CAR 14:0, CAR 24:1, Cer d18:1/16:0, Cer d18:2/18:0, Cer d18:1/24:1, and Cer d20:1/24:1 also associated with survival in univariate models; this single-center cohort has not yet been independently validated [151]. Also, deviated lipid concentrations above the usual standard track across prostate cancer’s development from localized to metastatic, as higher levels of ceramides alongside elevation of IL6, IL8, CXCL16, MPIF1, and YKL40 levels were noted, with ceramide baseline levels being a better predictor than cytokine measures of poor treatment response [152].

By 2026, lipidomics was longitudinally conducted with the plasma of 50 mCRPC patients undergoing enzalutamide treatment, with several key findings [153]. First, mCRPC patients had significantly elevated ceramides compared to cancer-free subjects at baseline, alongside enzalutamide treatment providing a stark reduction in ceramide concentration and an increase in sphingomyelins [153]. Androgen receptor inhibition can then be thought of as a balancer of the ceramide/SM metabolic axis. Finally, Sphingomyelin 18:1 post-treatment was an independent predictor of OS and progression-free survival after the researchers did multivariate adjustment [153]. Taken together, plasma lipidomics could eventually serve as a non-invasive monitoring tool for treatment response in advanced prostate cancer, but these results come from a single cohort of 50 patients and still require prospective validation [153].

### 10.5. Melanoma

Ceramides are major components of human skin; alongside free fatty acids and cholesterol, they compose a majority of the inter-corneocyte lipids while preventing water evaporation and pathogen entry [27]. A majority of human stratum corneum ceramide lengths range from 18 to 36, with 24–28 being most prevalent [154]. However, ceramide metabolism has been investigated for involvement in melanoma progression and dedifferentiation. Tumor necrosis factor alpha (TNF-α) impairs the anti-melanoma immune response in metastatic melanoma patients through induced cell death of activated CD8+ T lymphocytes, alongside resistance to anti-PD-1 antibodies in mice; TNF also promotes the epithelial-to-mesenchymal transformation (EMT), which is key for dedifferentiation [155]. This dedifferentiation furthers metastasis; in experimental models, TNF-α increased ceramide metabolite and glycosphingolipid accumulation, having a likely contributory effect on dedifferentiation [156]. Clinically, Dufau et al. profiled 78 plasma sphingolipids at baseline and week 6 in 48 patients with advanced melanoma receiving immune checkpoint inhibitors (ICI), looking for the most reliable predictors as levels evolved alongside therapy; the increase of the total amount of tri-hexosylceramides (CTH) was most predictive, and the next 3 best predictive features were also associated with glycosphingolipid metabolism and consisted of the increase of C24:0 di-hexosylceramide, C20:0 trihexosylceramide and C18:0 monohexosylceramide. After creating a sphingolipid score with the top 8 predictors to predict response to ICI, Dufau et al. found that a low sphingolipid score was significantly associated with better response (AUC 0.77) [156]. Because the score was developed and evaluated within the same cohort, it still needs validation in an independent cohort, which the authors plan in a dedicated trial [156].

AC is also overexpressed in normal melanocytes and proliferating melanoma cell lines compared to normal non-melanoma cells alongside fibroblasts and keratinocytes [157]. In biopsied samples with Stage II melanoma, high AC generation and expression were observed and specifically localized to the cytosol, in contrast to melanocytes, which possess cytosolic and nuclear AC and lower ceramide generation levels [157]. In order to test AC’s contribution to melanoma proliferation, AC inhibition through either ARN14988 (an AC inhibitor) or CRISPR-Cas9 ablation was found to increase ceramides and apoptosis, decrease S1P, and cause the same synergistic antitumoral agent (like doxorubicin) effect seen in other cancers undergoing AC inhibition [43,157,158,159]. These findings come from cell lines and tissue biopsies, identifying acid ceramidase as a therapeutic target rather than a circulating biomarker.

### 10.6. Multiple Myeloma and Hematological Cancers

Multiple myeloma (MM) is a currently incurable cancer impacting plasma cells of the bone marrow, with increased angiogenesis in the bone marrow with MM progression [160]. While proteasome inhibitors (e.g., bortezomib, carfilzomib, and ixazomib) alongside immunomodulatory drugs and CAR-T therapies have been used to selectively inhibit nuclear export of waste and improve patients’ prognoses, relapsed/refractory MM (RRMM) (where the disease progresses after treatment within 60 days of the given therapy) remains problematic and worsens patient prognosis [160,161]. Changes in MM microenvironment are being investigated as drivers of RRMM, spurring studies into metabolic reprogramming of the bone marrow microenvironment as a driver of resistance, with sphingolipid metabolism becoming a focus. Through an analysis of 672 patients’ RNA sequencing data, sphingolipid metabolic genes were found to have significantly higher expression in RRMM, with acid ceramidase ASAH1 being a key regulator of resistance to proteasome inhibitors [161,162]. Followed up by pharmacological and genetic knockout of ASAH1 in in vivo models, restoration of sensitivity to common MM drugs occurred [161]. Combined with the fact that patients with high expression of sphingolipid metabolic genes had worse OS alongside increased rates of RRMM, ASAH1 and other related enzymes are implicated as important targets. These data suggest that ASAH1 acts as a molecular switch converting therapeutically sensitive ceramide burdens into a survival signal; however, the link between sphingolipid-gene expression and worse outcomes comes from tumor RNA-sequencing data, so it represents tissue-based rather than circulating-biomarker evidence [161].

In preclinical models, combining the sphingosine kinase 2 (SPHK2) inhibitor ABC294640 with the Bcl-2 inhibitor ABT-199 (venetoclax) produced synergistic anti-myeloma effects in myeloma cell lines lacking the t(11;14) translocation [163]. This matters because ABT-199 activity in MM is concentrated in patients with t(11;14), whereas myeloma cells without the translocation rely more on the anti-apoptotic proteins Mcl-1 and Bcl-xL [163].

Through ABC294640 downregulating c-Myc and Mcl-1 simultaneously, as ABT-199 targets Bcl-2, the combination enhanced PARP and caspase-3/9 cleavage and increased apoptosis, even in the presence of bone marrow stromal cells, and suppressed tumor growth in a myeloma xenograft model more effectively than either drug alone [163]. These preclinical results suggest that sphingolipid-targeting combinations might extend Bcl-2 inhibitor therapy to patients without t(11;14), a possibility that still needs clinical evaluation.

The ceramide/S1P rheostat’s involvement in hematological malignancies has been explored, with the first link between sphingolipids and apoptosis occurring in HL-60 leukemic cells [164,165]. For AML, CML, and LGL leukemias as well as Gaucher disease and lymphomas, ceramides provided in combination with therapeutics can enhance anti-leukemic effects and increase druggability [164]. In AML specifically, acid ceramidase is upregulated and its inhibition restores ceramide-driven apoptotic sensitivity, similar to the ASAH1 mechanism in MM [166].

For the Gaucher disease-MM interaction, GBA1 deficiency leads to accumulation of glucosylceramide and glucosylsphingosine in plasma, with GBA1-deficient mice developing B cell lymphomas and monoclonal gammopathy, implicating ceramide glycosylation pathways’ involvement with lysosomal storage disorders for hematological malignancy risk [167]. Finally, Wątek et al. reported lower acid sphingomyelinase activity in dried blood spots from MM patients than from healthy controls, both at diagnosis and during treatment, whereas circulating ceramide, sphingosine, and sphinganine levels were higher in a small subset of patients [168]. Because ASMase activity did not differ significantly across ISS stages and the study lacked an independent validation cohort, ASMase activity remains an exploratory rather than an established monitoring marker [168].

### 10.7. Lung Cancer

Ceramide-focused therapeutic approaches have been discussed for thoracic and lung cancers [132]. When studying non-small cell lung cancer cell models, targeting glucosylceramide synthase induced antiproliferative and pro-apoptotic effects in osimertinib-resistant NSCLC cell models, restoring ceramide-driven apoptosis in cells that had developed resistance to 3rd-generation EGFR inhibition [132]. Glucosylceramide synthase inhibition thus holds promise to reverse therapeutic resistance in lung cancer alongside current treatments, although this has so far been shown only in cell models [132].

### 10.8. Therapeutics and Targeted Treatments with Ceramides

How can ceramides then, with their complex role within tumor biology, have meaningful clinical application for oncology patients? Ceramide-modulation approaches including inhibitors of ceramide-metabolizing enzymes, cell-permeable ceramide analogs, and ceramide-containing nanoparticles offer opportunities to resensitize tumors and increase treatment efficacy [130,131,134,136,169]. How GCS inhibitors restore pro-apoptotic ceramide levels in chemotherapy-resistant and multidrug-resistant tumors has been highlighted [42,131]. Acid ceramidase inhibitors also showed efficacy in reversing prostate cancer model drug resistance [43]. The comprehensive enzyme inhibitor framework laid out a strong suit of possibilities for cancer therapeutic developments: (1) increasing the ceramide to S1P ratio, (2) utilization of ceramides as mediators of the Bcl-2 family members, Akt, Myc, SR proteins, Rb, PKC, and JNK pathways, and (3) inhibition of acid ceramidase, sphingosine kinases, and GCS [41]. These are therapeutic rather than biomarker applications, and most remain at the preclinical stage. Figure 5 summarizes the strength of the clinical evidence for the cancers discussed in Section 10.

### 10.9. From Mechanism to Clinical Biomarker

Taken together, mechanistic and tissue studies consistently implicate ceramide metabolism, particularly the ceramide/S1P balance and the activities of GCS, acid ceramidase, and the sphingosine kinases, in tumor progression and therapy resistance. These findings justify therapeutic targeting, but they should not be read as evidence of diagnostic or prognostic utility. Circulating ceramide measurements have so far been evaluated mainly in small, single-center cohorts without independent validation (Figure 5). Prostate cancer is the exception, where PCPro was associated with shorter overall survival in two independent mCRPC cohorts and in a post hoc analysis of the ENZAMET trial in mHSPC; even there, the signature has not yet been tested prospectively as a guide to treatment. For the other cancers discussed here, ceramides remain candidate biomarkers rather than clinical tools.

## 11. Limitations of Existing Studies

Despite the growing field, interest, and literature in ceramides as clinical biomarkers, several key limitations currently impact the full utilization of ceramides in clinical work. First, there is no true clinical standardization of ceramide concentrations or cutoffs, with the CERT panels being the only cross-laboratory standard for comparison. Yet laboratories use different mass spectrometry protocols and programs; furthermore, the type of sample for lipid analysis often varies from lab to lab. For example, the Mielke et al. (2010) MCI study used plasma samples, but the Filippatou et al. MS study used serum alongside the IBD Elger study, but these different sample sources should not be directly compared [170,171]. For best clinical use, more comprehensive guidelines for how samples should be collected and analyzed for lipid and ceramide analysis must be developed to allow for better harmonization of ceramide concentrations for clinical use, allowing a “high” or “low” Cer(16:0) in one lab’s assay to be equivalent to another lab’s. Reducing the heterogeneity in methodologies across ceramide analysis and quantification in labs can better implicate ceramides in cardiovascular risk stratification.

The lack of standardized species-specific cutoffs for diseases other than cardiovascular disease in specific populations also limits the use of ceramides as a clinical tool. CERT and CERT2 [9] are specifically meant for adults at high risk for cardiovascular events, like diabetes mellitus, CAD or peripheral artery disease (PAD). For other diseases covered in this paper like IBD (Elger), COPD (Gharib et al., 2023), lupus (Hammad & Twal, 2021; Checa et al., 2017), RA (Shoghli et al., 2025), and neurodegeneration (Mielke et al., 2010), there exists no equivalent validated risk score, diagnostic profile or threshold in the literature or clinical practice, emphasizing the need for further work in the clinical potential of ceramides in a broad set of diseases [13,95,104,112,170,172,173,174].

A majority of associative ceramide-related studies are cohort or cross-sectional, with the FHS [8] and CHS [4] datasets being observational; the Elger IBD and lupus studies are cross-sectional. More interventional trial data with ceramide-focused treatments and modifications combined with outcome tracking is needed to distinguish ceramides from solely being markers [8,50].

Amidst these cohort populations, the majority of the studies focused on them are from predominantly Northern European or North American populations: FHS (Framingham, Massachusetts), CHS (4 US communities), FINRISK (Finland, Shoghli et al., 2025), and the Norwegian cohorts for Hilvo et al. (2020) [9,112]. Considering that ceramide metabolism and cardiovascular health are influenced by a host of diverse factors, including diet, environment, genetics, and gut microbiota, which all change substantially across different ethnicities and populations, it is important to be aware that the ceramide reference ranges and risk thresholds derived from these cohorts may not generalize globally.

Certain confounding factors are also crucial to note, specifically ceramide-disease associations in studies coming from populations with various concurrent conditions and specific drug use, like statins. Considering that the target population of cardiovascular risk-stratification tests is usually high-risk, previous cardiovascular event patients, it is important to acknowledge the impact of past diseases alongside potential statin treatment. Since statins impact sphingolipid metabolism, and high-CVD-risk patients in these cohorts often are statin-treated, this is a confounding factor alongside comorbidity that should be strongly considered.

Additionally, assay costs, turnaround time, laboratory accessibility, and cost-effectiveness can be barriers to routine clinical implementation. The cost to test ceramides can vary from $68–180 for clinical blood tests or over $300 for assays/ELISA kits [175]. Due to these high costs, it can be difficult to adopt ceramide testing as a standard procedure. To our knowledge, no formal cost-effectiveness analysis of ceramide testing has yet been published, and such data will be needed before routine adoption. Certain ceramide tests, such as the MI-Heart Ceramides Test, can have a turnaround time of 2–7 days [176]. This can delay time-sensitive diagnoses. Mayo Clinic Laboratories pioneered the commercialization of the MI-Heart Ceramides plasma assay; however, samples need to be collected and shipped to the clinic itself. This test is not widely available, leading to accessibility issues [176].

Finally, the gap between ceramides as a clinically used test and a research biomarker varies across diseases; while CVD has a stronghold of mature evidence with validated, commercially available risk-stratification tests (CERT1 and CERT2), IBD and COPD, for example, only have single preliminary studies like Elger et al. (2025) and Gharib et al. (2023), respectively [95,104]. For neurodegeneration, the Mielke et al. (2010) study is over 15 years old and has not been translated or followed up on for clinical use [170]. Answering the question “Are we there yet?” for clinical ceramide use for a diverse set of diseases with significantly different datasets and studies requires a more tailored, less general answer.

## 12. Perspectives and Future Directions

For ceramides to become clinically useful biomarkers of cardiovascular and other diseases, there are certain key directions and perspectives that must be accounted for.

First, studies other than observational or validation cohorts must be done, specifically randomized controlled trials where ceramides are directly modified and/or quantified in relation to risk stratification and treatment allocation alongside outcome tracking. Experimental studies focusing directly on the ceramides implicated in these observational studies are crucially needed; until that type of significant data exists, ceramides remain markers rather than utilizable clinical decision tools in how LDL cholesterol currently is.

Considering the vast number of ceramide analytical methodologies and lack of standardization, a similar standardization comparable to the work done for HbA1c standardization is needed, allowing for the same plasma samples to be run on different lipidomic platforms to establish conversion factors and universal reference ranges. This would transform the clinical ceramide literature into a collection of finally comparable studies.

For example, GCS inhibitors and ceramidase inhibitors have been described as ways to restore pro-apoptotic ceramide levels in chemotherapy-resistant tumors. The childhood cancer survivor study (Broberg et al., 2024) shows ceramide profiles remain altered years post-anthracycline therapy [136]. Through therapeutic validation strategies aimed at ceramides in different diseases, pharmacological reduction of long-chain ceramides (via CerS5/6 inhibition or SPT inhibition) demonstrably improving outcomes in metabolic or cardiovascular disease models would help provide the strongest evidence that ceramides are causal rather than associative.

Replicating the current CVD–ceramide literature, every non-CVD disease covered in this paper would benefit from a dedicated validation cohort study with pre-specified ceramide species and cutoffs, similar to CERT and CERT2. For example, when examining IBD and Elger et al.’s LC/VLC ratio, a replication with a larger, multi-site cohort with colonoscopic confirmation would greatly help establish ceramides’ potential as markers of IBD activity [104]. For COPD, Gharib et al.’s findings need replication in a non-elderly, more diverse cohort with pulmonary-specific metrics. For lupus, the Hammad & Twal (2021) and Checa et al. (2017) findings need more prospective validation tied to lupus-specific organ damage endpoints [13,172].

Glycosylated ceramides are increasingly becoming implicated in cardiac dysfunction in the ceramide literature base (Andersson et al. on GlcCer, Chatterjee et al. on LacCer, and Turner et al. 2024 on HLHS), with GlcCer and LacCer being significant in cardiac dysfunction [79,83,84]. Yet neither CERT nor CERT2 includes these species or any type of glycosylated ceramide, so an obvious future direction is testing whether combining glycosylated ceramide species with CERT2’s existing ceramide and a phospholipid framework improves discrimination, particularly for heart failure and ischemia-reperfusion outcomes.

Another area for exploration for application of ceramide biology into CVD prevention is through dietary and lifestyle modification. Recent clinical studies have shown that the ceramide profile is not fixed and can be modified by dietary composition [11,177,178]. A recent randomized study of patients at high cardiovascular risk on a Mediterranean diet for 28 months demonstrated improvement in their vascular profile. Furthermore, the levels of adipokines and individual ceramides, as well as the ceramide species ratios, were affected by diet [177]. In particular, saturated fatty acids, particularly long-chain saturated free fatty acids, a substrate for SPT (serine palmitoyltransferase), the rate-limiting enzyme for de novo ceramide synthesis, can increase the levels of individual ceramides and ceramide species ratios that affect cardiovascular disease risk, including CERT2 -related species ratios [11,178]. On the other hand, unsaturated free fatty acids, particularly polyunsaturated fatty acids, a component of the Mediterranean diet, can decrease the levels of individual ceramides and the ceramide species ratios that increase cardiovascular disease risk, including CERT2-related ceramide species ratios [11,178]. Therefore, fat composition in the diet could be a crucial determinant of the levels of individual ceramides and ceramide species ratios that determine cardiovascular disease risk [11,178]. Studies have shown that Mediterranean diets, as well as other dietary approaches to the treatment of cardiovascular diseases such as calorie restriction and certain fiber and polyphenol consumption, can decrease the levels of individual ceramides and ceramide species ratios that determine cardiovascular disease risk, including CERT2-related ceramide species ratios [178]. To solidify evidence for sustained alteration of CERT/CERT2-related ceramide profiles and their translation into decreased cardiovascular disease events, future trials will be needed [177,178].

For lifestyle modification: exercise, physical activity, and bed rest have been studied to understand impact on ceramide levels [179]. Short- and long-term bed rest invokes the skeletal muscle and circulatory systems’ response, with glycogen storage after short-term bed rest resulting in increased insulin sensitivity but decreased cell membrane GLUT4 [180,181]. This minimizes glycogen buildup, highlighting the temporal relationship between ceramides, sphingomyelins, and triglycerides with skeletal muscle’s reduced metabolic functioning occurring after long-term bed rest [180,181]. This relationship was further extended by analyzing time-related changes in circulating ceramides in 35 relatively healthy young and older adults after 5 days of bed rest; CERT1 and C18:0/C24:0 ceramide ratios were increased alongside their inversely related biomarker counterparts, demonstrating lowered adiponectin, FGF21, and physical performance [182]. The results point to a method for future detection of CV dysregulation and dysfunction resulting from short-term inactivity because of aging. In the 29 Baltimore Longitudinal Study of Aging (BLSA) participants, blood samples were collected before, after exercise, and during recovery. Participants were classified as low- or high-fitness; the high-fitness groups showed a decrease in 11 distinct ceramides, and 8 for the low-fitness counterpart. With the recovery stage possessing the most significant reductions, the baseline ceramide profiles for both groups were rather similar, with the exception of Cer d18:0/24:1’s higher concentration in low-fitness groups. But as recovery progressed to the one-hour mark, Cer d18:1/26:0, Hex3-Cer d18:1/16:0, and Cer d18:0/24:1 differed significantly between the high- and low-fitness individuals, pointing to baseline conditioning’s impact on regulation of lipid profiles [183].

Finally, as CERT2’s improved performance over CERT1 (Hilvo et al., 2020) arose from replacing several ceramide measures with ceramide/phosphatidylcholine ratios and a phosphatidylcholine concentration, this should emphasize the need for multi-omic integration of different classes of lipids alongside ceramides [9,184]. TMAO/PC work (Wang et al., 2011) and the lupus lipidomics studies (Koelmel et al., 2026; Sun et al., 2022) both pointed toward sphingolipid profiles being most useful and informative when analyzed next to other lipid classes and inflammatory markers, highlighting that future work should test whether ceramide/phospholipid/inflammatory cytokine panels or ceramide/microbiome composition models can outperform ceramide ratios alone [12,87,124]. This is particularly important for weak ceramide biomarker performance for non-CVD diseases with few dedicated lipid panels or ceramide studies.

## 13. Conclusions

Across cardiovascular, metabolic, inflammatory, and oncological diseases, ceramides have emerged as an informative lipid biomarker. Returning to the questions we began with, the strongest and most clinically ready evidence lies in cardiovascular disease, where specific ceramides and, more importantly, their ratios, such as Cer(16:0)/Cer(24:0), have been shown to produce robust and reproducible associations with disease progression and major cardiovascular events, often beyond LDL cholesterol and traditional risk factors. The development and clinical implementation of scoring systems such as CERT and CERT2 further reinforce the translational potential of ceramides, particularly when combined with other lipid species and traditional risk factors. In this context, ceramides represent a validated addition to cardiovascular risk stratification and prognostic frameworks, rather than a diagnostic test.

Beyond cardiovascular disease, the role of ceramides remains more exploratory. In metabolic diseases such as type 2 diabetes, ceramides function as integrative markers of insulin resistance and lipotoxic stress, reinforcing their role in linking metabolic dysfunction to cardiovascular risk. In conditions such as IBD, cancer, COPD, and AIRDs, ceramide dysregulation is consistently observed, but its clinical utility is less clearly tested and defined (Figure 4 and Figure 5). Across all of these diseases, a consistent pattern emerges: ceramides are most informative when evaluated within a broader lipidomic framework rather than as isolated species measurements.

So, are we there yet? For cardiovascular risk prediction and prognosis, largely yes, as an adjunct to established risk tools; for the other diseases covered here, not yet, and their implications necessitate far more thorough interventional and cohort work. Simultaneously, ceramides should undergo the same standardization process cholesterol did in the 1980s–90s, with better methodological and quantitative protocols, so that ceramides’ clinical use and the research around them improve.

## Figures and Tables

**Table 1:** The six mammalian ceramide synthase (CerS) isoforms, their preferred fatty acyl-chain lengths, the ceramide species they produce, and their reported biological and prognostic associations.

CerS Isoform	Preferred Acyl Chain	Main Ceramide Products	Chain-Length Class	Reported Associations
CerS1	C18:0	Cer(18:0)	Long-chain	Mitochondrial dysfunction, insulin resistance, endothelial apoptosis, and pro-atherogenic inflammation [2,18]
CerS2	C22:0–C24:0	Cer(22:0), Cer(24:0)	Very-long-chain	Structurally and functionally protective; contributes to membrane integrity and may mitigate the lipotoxic effects of shorter-chain ceramides [8,18,23]
CerS3	≥C26:0	Ultra-long-chain ceramides	Ultra-long-chain	Expressed mainly in skin and testis; essential components of the epidermal permeability barrier [18,27]. Increased activity raised Cer(d18:1/26:0), activating EGFR and colorectal cancer progression (Section 10.3) [28]
CerS4	C18:0–C20:0	Cer(18:0), Cer(20:0)	Long-chain	Broadly expressed, high levels in skin, heart and leukocytes [18]; in colorectal cancer, lower in KRAS-mutant than KRAS-wild-type tumors, consistent with lower tumor C18:0 and C20:0 ceramides; associated with vascular invasion (Section 10.3) [29,30]
CerS5	C14:0–C16:0	Cer(16:0)	Long-chain	Mitochondrial dysfunction, insulin resistance, endothelial apoptosis, and pro-atherogenic inflammation [2,18,22]
CerS6	C14:0–C16:0	Cer(16:0)	Long-chain	Mitochondrial dysfunction, insulin resistance, endothelial apoptosis, and pro-atherogenic inflammation [2,18,22]

**Table 2:** Composition, scoring, study populations, and endpoints of the ceramide-based cardiovascular risk scores CERT1 and CERT2.

	CERT1	CERT2
Full name	Coronary Event Risk Test	Cardiovascular Event Risk Test 2
Original report	Laaksonen et al., 2016 [6]	Hilvo et al., 2020 [9]
Lipid components	Cer(d18:1/16:0), Cer(d18:1/18:0), and Cer(d18:1/24:1) concentrations, and each of these divided by Cer(d18:1/24:0)	Cer(d18:1/24:1)/Cer(d18:1/24:0); Cer(d18:1/16:0)/PC(16:0/22:5); Cer(d18:1/18:0)/PC(14:0/22:6); PC(16:0/16:0) concentration
Absolute vs. ratio measures	3 concentrations + 3 ratios	3 ratios + 1 concentration
Scoring	Quartile-based: 1 point for the 3rd and 2 points for the 4th quartile of each component; total 0–12 [31]	Quartile-based points for each component; total 0–12 [9,31]
Risk categories	Low (0–2), moderate (3–6), increased (7–9), high (10–12) [31]	Low (0–3), moderate (4–6), increased (7–8), high (9–12) [31]
Clinical covariates	None	None in the lipid score; age and sex in the extended prognostic model [9]
Study populations	Stable CAD and ACS cohorts [6]; FINRISK 2002 primary-prevention cohort (*n* = 8101) [31]	Derivation: WECAC, stable CAD (*n* = 3789); validation: LIPID (*n* = 5991) and KAROLA (*n* = 1023) [9]; further evaluated in hemodialysis (AURORA, 4D) [48] and in statin- and PCSK9 inhibitor-treated patients [49]
Main endpoints	Cardiovascular death	Cardiovascular death; composite of cardiovascular death, MI, and stroke
Reported performance	Relative risk of CV death, high vs. low category: 4.2 (stable CAD) and 6.0 (ACS); HR per SD for CV death 1.27 in WECAC [31]	C-index with age and sex (WECAC): 0.76 for CV death and 0.65 for composite events [9]; HR per SD for CV death 1.50 (WECAC), 1.51 (LIPID), 1.62 (KAROLA) [31]; C-index 0.59–0.60 in hemodialysis [48]

ACS, acute coronary syndrome; CAD, coronary artery disease; CV, cardiovascular; HR, hazard ratio; KAROLA, Langzeiterfolge der KARdiOLogischen Anschlussheilbehandlung; LIPID, Long-Term Intervention with Pravastatin in Ischaemic Disease; MI, myocardial infarction; PC, phosphatidylcholine; SD, standard deviation; WECAC, Western Norway Coronary Angiography Cohort.

## Data Availability

No new data were created or analyzed in this study. Data sharing is not applicable to this article.

## Supplementary Materials

The following supporting information can be downloaded at: https://www.mdpi.com/article/10.3390/ijms27219605/s1.

## Abbreviations

The following abbreviations are used in this manuscript:

4D	Die Deutsche Diabetes Dialyse Studie
AC	acid ceramidase
ADT	androgen deprivation therapy
AIRD	autoimmune rheumatic disease
AR	androgen receptor
ASCVD	atherosclerotic cardiovascular disease
ASMase	acid sphingomyelinase
AUROC	area under the receiver operating characteristic curve
AURORA	A Study to Evaluate the Use of Rosuvastatin in Subjects on Regular Hemodialysis: An Assessment of Survival and Cardiovascular Events
BLSA	Baltimore Longitudinal Study of Aging
BMI	body mass index
CAD	coronary artery disease
CD	Crohn’s disease
Cer	ceramide
CerS	ceramide synthase
CERT	Coronary Event Risk Test (also CERT1)
CERT2	Cardiovascular Event Risk Test 2
CHD	coronary heart disease
CHS	Cardiovascular Health Study
CKD	chronic kidney disease
COPD	chronic obstructive pulmonary disease
CRC	colorectal cancer
CRP	C-reactive protein
CTH	trihexosylceramide
CVD	cardiovascular disease
ESKD	end-stage kidney disease
EV	extracellular vesicle
FHS	Framingham Heart Study
FINRISK	Finnish population-based cohort study (National FINRISK Study)
GCS	glucosylceramide synthase
GlcCer	glucosylceramide
HbA1c	hemoglobin A1c
HDL-C	high-density lipoprotein cholesterol
HFpEF	heart failure with preserved ejection fraction
HIIT	high-intensity interval training
HLHS	hypoplastic left heart syndrome
IBD	inflammatory bowel disease
ICI	immune checkpoint inhibitor
ISS	International Staging System
LacCer	lactosylceramide
LC	long-chain
LDL-C	low-density lipoprotein cholesterol
LN	lupus nephritis
LVEF	left ventricular ejection fraction
MACE	major adverse cardiovascular events
MASH	metabolic dysfunction-associated steatohepatitis
MCI	mild cognitive impairment
mCRPC	metastatic castration-resistant prostate cancer
mHSPC	metastatic hormone-sensitive prostate cancer
MI	myocardial infarction
MM	multiple myeloma
NAFLD	non-alcoholic fatty liver disease
NASH	non-alcoholic steatohepatitis
OS	overall survival
PAD	peripheral artery disease
PC	phosphatidylcholine
PCSK9	proprotein convertase subtilisin/kexin type 9
PCSK9i	PCSK9 inhibitor
PSA	prostate-specific antigen
RA	rheumatoid arthritis
RRMM	relapsed/refractory multiple myeloma
S1P	sphingosine-1-phosphate
SCA	sudden cardiac arrest
SCD	sudden cardiac death
SCORE2	Systematic Coronary Risk Evaluation 2
SLE	systemic lupus erythematosus
SLEDAI	Systemic Lupus Erythematosus Disease Activity Index
SM	sphingomyelin
SMase	sphingomyelinase
SMPD3	sphingomyelin phosphodiesterase 3
SPHK	sphingosine kinase
SPT	serine palmitoyltransferase
TC	total cholesterol
TG	triglyceride
TMAO	trimethylamine N-oxide
UC	ulcerative colitis
UGCG	UDP-glucose ceramide glucosyltransferase
VLC	very-long-chain
WECAC	Western Norway Coronary Angiography Cohort
