# Supplementary material for: Ceramides as Biomarkers of Risk Prediction and Prognosis in Cardiovascular and Other Diseases: Are We There Yet?
Source: Int J Mol Sci. 2026 Oct 27;27(21):9605. doi: 10.3390/ijms27219605 (PMC13763435; doi:10.3390/ijms27219605)
Supplement: Supplementary file 1 [file ijms-27-09605-s001.zip › ijms-4632588-supplementary.pdf]

Supplementary Materials

Section S1. Literature search strategy

Table S1. Literature search strategy by disease category, with the number of records retrieved and the number of articles included (n). Disease-category searches were run in PubMed and Scopus for January 1990 to June 2026; the background and historical search had no date limit. Each included article is counted once, in the category in which it is cited most often; sources cited only in the general sections (Sections 1, 3 and 11–13) are counted under Background.

Disease category	Search strings (one per line)	Records retrieved	Articles included (n)
Cardiovascular Disease & Ceramide Biomarkers	ceramide AND (cardiovascular disease OR coronary artery disease OR heart failure OR atherosclerosis OR MACE)<br>(CERT1 OR CERT2) AND cardiac risk	2,480	18
CVD Risk Prediction & CERT Scores	ceramide AND (LDL OR risk stratification)<br>ceramide AND (CERT OR risk score OR predictive marker)<br>(Cer(d18:1/16:0) OR Cer(d18:1/24:0)) AND cardiovascular	789	20
Heart Failure & Cardiomyopathy	ceramide AND (heart failure OR cardiomyopathy OR ejection fraction OR systolic dysfunction)	406	4
Autoimmune Rheumatic Diseases (AIRDs)	ceramide AND (autoimmune OR rheumatic disease OR AIRD)<br>sphingolipid AND inflammation AND lipid metabolism	1,440	4
Rheumatoid Arthritis	ceramide AND rheumatoid arthritis<br>CERT AND RA<br>sphingolipid AND joint inflammation	328	10
Systemic Lupus Erythematosus	ceramide AND (lupus OR SLE OR systemic lupus erythematosus)<br>sphingolipid AND autoantibody<br>Cer(16:0)/S1P AND lupus	1,299	9
Metabolic Disease	ceramide AND (diabetes mellitus OR insulin resistance OR obesity OR metabolic syndrome)<br>dihydroceramide AND glucose homeostasis<br>sphingolipid AND adipose tissue	2,541	1
Type 2 Diabetes & Insulin Resistance	ceramide AND (type 2 diabetes OR insulin resistance OR hyperglycemia)<br>ceramide AND pancreatic beta cell	1,646	5
Non-alcoholic Fatty Liver Disease	ceramide AND (NAFLD OR NASH OR hepatic steatosis OR liver fibrosis)	940	6
Cancer	ceramide AND (cancer OR tumor OR apoptosis OR chemotherapy resistance)<br>nanoliposomal ceramide AND oncology<br>ceramide AND (colorectal OR breast OR lung OR ovarian OR myeloma OR prostate cancer)	10,699	48
Therapeutic Resistance & Ceramide Metabolism	glucosylceramide synthase AND chemotherapy resistance<br>alkaline ceramidase AND tumor survival<br>sphingolipid AND drug resistance	1,948	3

Disease category	Search strings (one per line)	Records retrieved	Articles included (n)
Inflammatory Bowel Disease	ceramide AND (Crohn's disease OR ulcerative colitis OR inflammatory bowel disease)<br>sphingolipid AND intestinal epithelial barrier<br>ceramide AND gut microbiome	271	6
COPD & Pulmonary Disease	ceramide AND (COPD OR chronic obstructive pulmonary disease OR emphysema OR pulmonary inflammation)<br>sphingolipid AND airway epithelium<br>ceramide AND smoking	458	10
Chronic Kidney Disease / ESKD	ceramide AND (chronic kidney disease OR CKD OR end-stage kidney disease OR ESKD OR hemodialysis)<br>CERT2 AND (hemodialysis OR ESKD)<br>ceramide AND (AURORA OR 4D)	150	3
Background, Historical & Cross-cutting (not disease-specific)	Targeted searches and citation tracking for the historical and biochemical sources cited only in Sections 1, 3 and 11–13 (no date limit)	131	34
**Total**		**25,526**	**181**

Section S2. Study selection

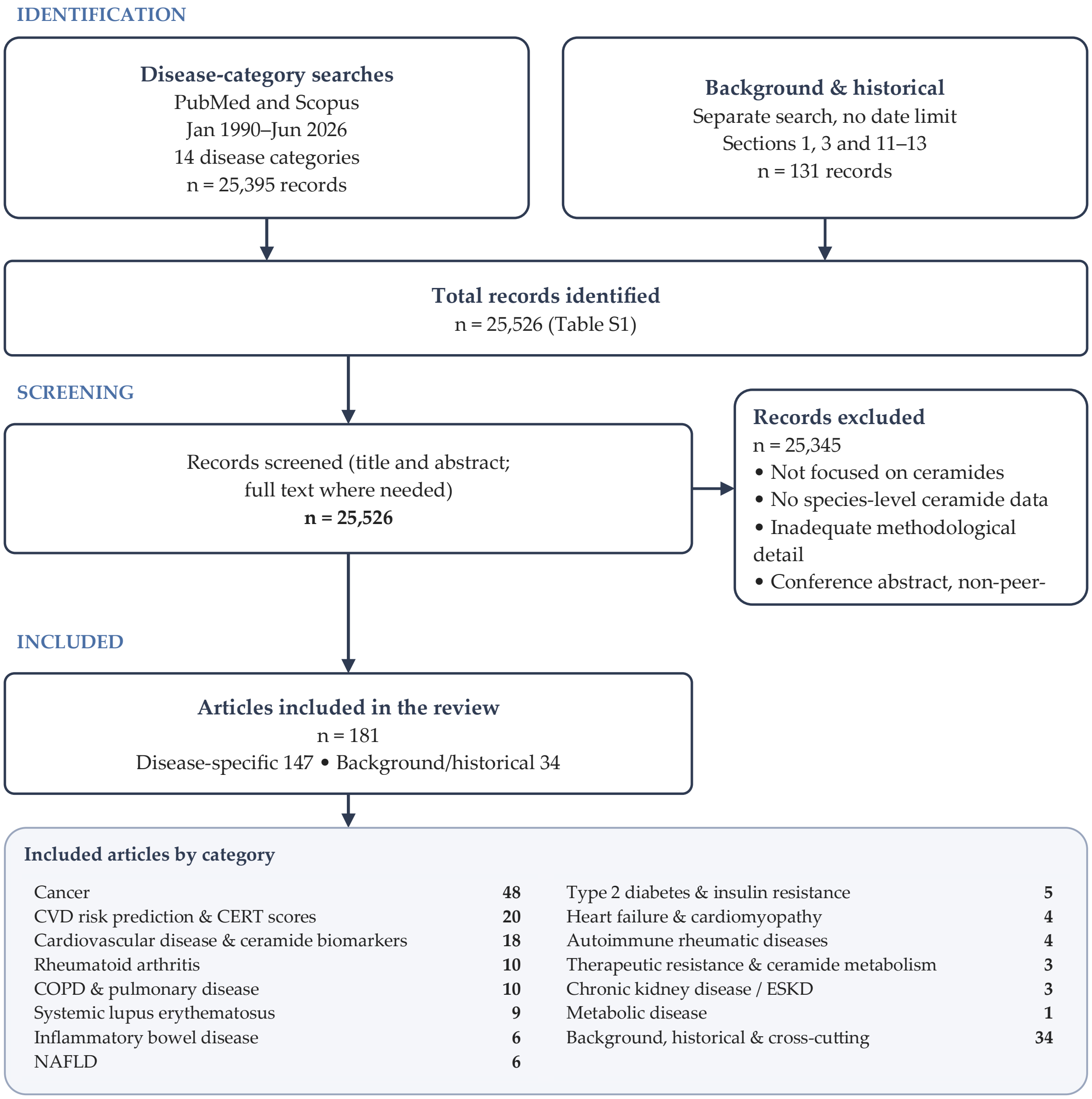

Figure S1. Flow diagram of study identification, screening and inclusion. The disease-category searches covered January 1990 to June 2026; the background and historical search (Sections 1, 3 and 11–13) had no date limit. Excluded records include those retrieved by more than one category search. Each included article is counted once, in the category in which it is cited most often. This is a narrative review; the diagram is provided for transparency and does not denote a PRISMA-compliant systematic review.
